# Energized Oxygen in the Magnetotail: Onset and Evolution of Magnetic Reconnection

**DOI:** 10.1029/2020JA028381

**Published:** 2022-09-26

**Authors:** Don E George, Jörg‐Micha Jahn

**Affiliations:** ^1^ Space Science and Engineering Southwest Research Institute San Antonio TX USA; ^2^ Department of Physics and Astronomy University of Texas at San Antonio San Antonio TX USA

**Keywords:** magnetotail, magnetic reconnection, energized oxygen, particle‐in‐cell simulation

## Abstract

Oxygen ions are a major constituent of magnetospheric plasma, yet the role of oxygen in processes such as magnetic reconnection continues to be poorly understood. Observations show that significant amounts of energized O^+^ can be present in a magnetotail current sheet (CS). A population of thermal O^+^ only has a relatively minor effect on magnetic reconnection. Despite this, published studies have so far only concentrated on the role of the low‐energy thermal O^+^. We present a study of magnetic reconnection in a thinning CS with energized O^+^ present. Well‐established, three‐species, 2.5D particle‐in‐cell (PIC) kinetic simulations are used. Simulations of thermal H^+^ and thermal O^+^ validate our setup against published results. We then energize a thermal background O^+^ based on published in situ measurements. A range of energization is applied to the background O^+^. We discuss the effects of energized O^+^ on CS thinning and the onset and evolution of magnetic reconnection. The presence of energized O^+^ causes a two‐regime onset response in a thinning CS. As energization increases in the lower‐regime, reconnection develops at a single primary *X*‐line, increases time‐to‐onset, and suppresses the rate of evolution. As energization continues to increase in the higher‐regime, reconnection develops at multiple *X*‐lines, forming a stochastic plasmoid chain; decreases time‐to‐onset; and enhances evolution via a plasmoid instability. Energized O^+^ drives a depletion of the background H^+^ around the central CS. As the energization increases, the CS thinning begins to slow and eventually reverses.

## Key Points

- Energized O^+^ has a major impact on the onset and evolution of magnetic reconnection
- The presence of energized O^+^ causes a two‐regime onset response in a thinning current sheet
- At lower energization, O^+^ increases time‐to‐onset and suppresses the rate of evolution; at higher energization, the opposite occurs

## 1 Introduction

### 1.1 Background

Magnetic reconnection plays a vital role in the behavior of magnetized plasmas in Earth's magnetosphere, the Sun, magnetically confined plasmas, and across the Universe. Understanding reconnection as a fundamental physical process is already a high priority of the space physics community. It is the primary science objective of the Magnetospheric Multiscale (MMS) Mission launched in March 2015. O^+^ is present throughout the magnetosphere in varying quantities. While heavy ions such as oxygen are known to influence the reconnection process in the Earth's magnetosphere, their influence remains one of the many aspects of magnetic reconnection that is poorly understood. There is no agreement on the degree to which, or precisely how, heavy ions affect the dynamics of magnetic reconnection. In addition, despite the presence of energized O^+^ along with thermal energies, simulation studies of reconnection have been conducted exclusively with thermal heavy ions, neglecting energized heavy ions.

### 1.2 Current Sheet Thinning to Onset—Expected Behavior

Research on the theory, observation, and simulation of magnetic reconnection in laboratories and in space has a long history. Zweibel and Yamada (2016), Pontin (2020), and their cited references provide excellent overviews of the history and recent understanding of magnetic reconnection.

Setting aside specific models and conditions, one can describe the basic evolution of magnetic reconnection beginning in the context of a simplified thinning current sheet (CS). Note that such a simplified description does not include all phenomena present in a physical reconnection system. A simplified antiparallel magnetic field system, such as the configuration used here, allows for a comparison of variations in individual parameters such as composition, energization, density, and CS thickness. Our basic configuration does not include guide fields (*B*_*y*_) or major *B*_*z*_ components.

Dayside reconnection, between the closed magnetic field lines of the Earth and those of the Sun, produces regions of open field lines connecting the Earth and Sun. With one end fixed at the Earth, these field lines are convected past the Earth by the solar wind, and accumulate in the lobes of the magnetotail. The two lobe regions of “frozen‐in” magnetized plasma “wrap” around the Earth and meet anti‐Sunward to form the magnetotail. The frozen‐in condition existing in these lobes prevents the magnetic field lines in these regions from merging. Where the lobes press against one another, a thick boundary forms, which is called the plasma sheet or layer. Embedded within the plasma sheet a CS forms along the magnetic reversal (neutral) between the lobes. Duskward currents of charged particles move in opposite directions (ions duskward and electrons dawnward) perpendicular to the magnetic field lines. The structure of this CS provides an additional barrier to the merging of the two regions. The build‐up of plasma and flux in the lobes “compresses” the CS, causing an increase in the external to internal pressure. Generally, this forces thinning of the CS over time and leads to conditions favorable to magnetic reconnection. Additionally, conditions may arise in which a degradation and disruption of the CS also leads to conditions favorable to magnetic reconnection.

We present a description of the expected behavior of a thinning and reconnecting CS using the features and terminology of the magnetotail. It is intended to aid in the evaluation of our simulations by providing features for comparing the evolution. Earth's magnetotail falls into the general category of a collisionless plasma based on the plasma parameters and system size. Ultimately, these parameters rely on the plasma ion content and magnetic field conditions in and around the CS. These conditions cause magnetic reconnection to develop in one of two configurations; with a single *X*‐line or with multiple *X*‐lines (Ji & Daughton, 2011). Note that Ji and Daughton (2011) intentionally avoid addressing the causes and configurations associated with the onset of magnetic reconnection. They state that the parameter regimes for onset are likely very different than those of the development of reconnection as addressed in their work. This depends on where the effective plasma size lies in terms of a critical transition size *λ*_crit_. Those systems with *λ* < *λ*_crit_ evolve with a single *X*‐point, while systems with *λ* > *λ*_crit_ evolve with multiple *X*‐points. While not based on analytical theory, a *λ*_crit_ ≈ 50 is empirically derived (Ji & Daughton, 2011).

The thickness of the central CS is governed by the balance of its internal pressure with both magnetic and plasma pressure from the bulk (lobe) regions above and below it. Thinning occurs due to the external pressure from the lobes as flux and plasma build up around the central CS. As the CS thins, instabilities form, initiating localized fluctuations. Once appropriate dissipative conditions are present, field lines from opposite lobes merge, altering the topology, and releasing previously frozen‐in energy. The field lines in the magnetotail lobes are open, i.e., connected from the Earth to the solar wind (the Sun). Once tail field lines have reconnected, they are closed, i.e., connected Earth to Earth.

Two‐dimensional magnetic reconnection can commence (“reach onset”) by various instability mechanisms. Once onset has occurred, the resulting reconnection configurations have been organized into a “phase diagram” based on two key dimensionless parameters (Ji & Daughton, 2011). These parameters govern which instability becomes dominant and how a system undergoing magnetic reconnection will evolve. First is the Lundquist number, *S* = 4*πν*_*A*_ *L*_*sp*_/*ηc* ^2^, where *ν*_*A*_ is the Alfvén velocity and *η* is the plasma resistivity. Second is the scaled macroscopic system size, *λ* = *L*_*sp*_/*d*_*i*_ (not to be confused with the collisional mean‐free path *λ*), where *L*_*sp*_ is the half length of the system and *d*_*i*_ is the proton inertial length (Cassak et al., 2005). Depending on the value of these two parameters, magnetic reconnection in the magnetotail can reach onset via single or multiple *X*‐line generation. However, it is important to note that plasma in the magnetotail is collisionless such that, values of the Lundquist number, *S*, are extremely large. This leaves either the system size or some other, as yet unestablished parameter, in control of the evolution of magnetic reconnection being in the form of a single *X*‐line or multiple *X*‐lines. Additionally, those parameters controlling the onset of reconnection are still to be determined and studied.

**Single X‐line:** Energy from the magnetic field reconfiguration accelerates the previously frozen‐in plasma out of the reconnection region. Additionally, this release of magnetic tension caused by the new topology of the field lines carries the reconfigured magnetic flux away from the reconnection region, lowering the pressure. This causes the higher pressure plasma in the lobes to “feed” into the reconnection region.

When studied in a 2.5D (2D spatial and 3D fields and particle velocities) context, the magnetic reconnection region produces several characteristic signatures in its evolution.

- A magnetic field *X*‐line, at the center of the reconnection region where thinning has occurred.
- Along the *X*‐line, electron‐ion decoupling (Hall effect) takes place at the scale length of ions (e.g., protons or O^+^) (Birn & Priest, 2007; Zweibel & Yamada, 2016). This generates an out‐of‐plane quadrupole Hall magnetic field (*B*_*y*_) around the *X*‐point. Additionally, off‐diagonal components of the electron pressure tensor (nongyrotropic) contribute to an out‐of‐plane electric field (*E*_*y*_) (Hesse et al., 1999). This is referred to as the reconnection electric field.
- Bulk inflow occurs, from the regions of magnetized plasma above and below the CS (lobes), toward the *X*‐line.
- Outflow jets of streaming ions, electrons, and magnetic flux flow away from the *X*‐line perpendicular to current flow in the CS.

**Multiple X‐line:** Compared to the overall amount of studies done on 2D reconnection, the study of the onset of magnetic reconnection leading to the formation of plasmoids is still in its growth phase. The authors are currently unaware of any published studies on the effect of energized heavy ions on the evolution of plasmoid chains. Karimabadi et al. (2011) does address plasmoid development in the presence of thermal background oxygen but not involving energized O^+^.

Multiple *X*‐lines may form producing “plasmoids” also called magnetic “islands” in 2D (also “flux ropes” in 3D) in the CS. We use islands to refer to smaller features that do not drive system evolution as a primary *X*‐point would. We use plasmoids to refer to large structures that dominate the CS.

Secondary CSs form between plasmoids. Secondary tearing instabilities trigger additional magnetic reconnection that causes plasmoids to coalesce. Over time, multiple levels of secondary reconnection can occur. In each level, a different scale size leads to variations in the local Lundquist number, resulting in varied local rates of reconnection. This causes the amount of reconnected magnetic flux to increase at a rate faster than in single *X*‐line reconnection.

Focusing on the initial CS thickness and using fully kinetic simulations, Daughton et al. (2009) found that for magnetic reconnection generating a plasmoid chain, the number of plasmoids, *N*, increased with the Lundquist number as *S* ^0.6^. This trend agrees with magnetohydrodynamic (MHD) theory by Loureiro et al. (2007) who found *N* increases as *S* ^3/8^. Additionally, Daughton et al. (2009) found that the time‐to‐onset decreases with an increasing Lundquist number as *S* ^−0.5^. Comisso et al. (2016) presented a general theory of plasmoid instability including the effects of an initial perturbation amplitude, the characteristic rate of CS evolution and the Lundquist number (*S*). They stress that the scaling relationships for a system evolving to plasmoid instability are not simple power laws. They found that the manner in which a thinning CS evolves, its onset time, and the manner in which magnetic reconnection proceeds are all according to a “principle of least time.” Neglecting the less interesting physics, i.e., that of a system evolving in a linear regime, they found that an initial perturbation, the characteristic rate of CS evolution and the Lundquist Number, *S*, determine the time‐to*onset, rate of evolution, and number of plasmoids produced. They go on to explain that a system remains quiescent, until it thins to a critical value, at which time the plasmoid instability occurs causing explosive growth of the plasmoids. One of their key findings indicates that above some critical Lundquist number, *S*_*c*_, a plasmoid instability becomes dominant and disrupts the CS while below *S*_*c*_ the plasmoid instability cannot grow before a single tearing instability forms and dominates the CS. The Comisso et al. (2016) plasmoid chain model was confirmed through MHD simulations where the Lundquist number has exceeded a critical value (Baty, 2020). However, below such a critical value, at low Lundquist numbers, a single *X*‐line can form under appropriate conditions.

Overall, the discussion above indicates that a thinning CS could evolve in one of two ways, either, at low *S* with a single *X*‐point becoming dominant or beyond the critical Lundquist number *S*_*c*_, with a plasmoid chain via a secondary tearing (plasmoid) instability. Up to this point, this behavior has not been studied or seen in studies involving heavy ions.

### 1.3 Thermal Oxygen and Magnetic Reconnection

Until now, simulations of magnetic reconnection involving oxygen have focused exclusively on a uniform thermal oxygen background (Hesse & Birn, 2004; Karimabadi et al., 2011; Liang et al., 2016, 2017; Markidis et al., 2011; Tenfjord et al., 2019). Oxygen has been treated the same as larger protons, which produce the same behavior, only on a larger scale. Background thermal oxygen has limited effects on the overall evolution of reconnection, simply acting as a “bigger” proton, maintaining about the same effects. The higher mass directly affects the Alfvén speed leading to the two primary effects of thermal O^+^ on reconnection. First, thermal oxygen scales the structure of the reconnection region to a physically larger size while maintaining the same diffusion region aspect ratio. Second, it appears to slow the rate of reconnection, although some have reported that heavy ions increase the reconnection rate.

Winglee (2004) indicates that in his global multifluid modeling of the magnetotail, the heavy ions introduce an inherently larger‐scale length to the system. The ion cyclotron scale is important to the dynamics of the magnetosphere, especially the presence of the heavy ionospheric ions. Since heavy ions become the first to demagnetize, they are critical to the formation of the diffusion region around the reconnection region. In addition, localized density enhancements and depletions are seen in the tail where the local heavy ion density can be substantially elevated. Because of these local density variations, reconnection across the tail is inhomogeneous. Winglee (2004) deals with global magnetotail dynamics as opposed to local reconnection system behavior. This increase in system scale is attributed to the increased mass, which enhances the changes in gyroradius and Alfvén speed.

Wiltberger et al. (2010) states that the O^+^ of ionospheric origin changes the reconnection rate as evidenced by a 6‐min delay in the final release of the first plasmoid. The reconnection rate is proportional to the Alfvén speed in the fluid flowing into the reconnection diffusion region. Therefore, a reduction in the reconnection rate is expected in the simulation with [ionospheric] outflow owing to the reduction in Alfvén speed caused by the O^+^ ions in the lobe inflow region. Tenfjord et al. (2019) found that the presence of thermal O^+^ (or other heavy ions) significantly decreases the reconnection rate, while the temperature (1.0 and 0.2 keV) has no significant effect. One common result of the presence of O^+^ in collisionless reconnection is the increased scaling of the quadrupole structure and dimensions of the diffusion region; however, the effect on the reconnection rate is not clear (Liu et al., 2015). Acceleration of thermal O^+^, due only to the reconnection electric field and not external to the reconnection process was noted (Karimabadi et al., 2011; Liu et al., 2015).

Multiple studies have touched on the effects of heavy ions on the rate of reconnection in the magnetosphere: Shay et al. (2007) using a 2D, 3‐fluid model; Markidis et al. (2011) using a 2.5D Particle‐In‐Cell (PIC); and Karimabadi et al. (2011) using a 2D PIC simulation. Shay et al. (2007) and Karimabadi et al. (2011) reported a marked decrease in the rate of magnetic reconnection due specifically to the presence of oxygen in the inflow region. Hesse and Birn (2004) concluded that background oxygen does not strongly restrict the reconnection rate. Markidis et al. (2011), Hesse and Birn (2004), and Karimabadi et al. (2011) reported that the presence of an O^+^ population slightly decreases the reconnection rate.

Baker et al. (1982) and Liu et al. (2013) argue from observational data that O^+^ may be increasing the reconnection rate. While Baker et al. (1982) notes that enhanced densities of O^+^ may define regions where explosive reconnection could be initiated, Liu et al. (2013) states that their results suggest that a higher O^+^ content makes it more difficult to trigger reconnection. Although most of the evidence indicates slowing, there is still some disagreement as to how the presence of heavy ions affects the onset of reconnection as well as the reconnection rate.

### 1.4 Energized Oxygen

Although observed in situ (Kistler et al., 2005), magnetic reconnection in the presence of an *energized* O^+^ background has not previously been investigated via kinetic PIC simulations. Other than studies of acceleration produced by magnetic reconnection itself, and our work on energized O^+^ bifurcation (George & Jahn, 2020), no previous work has performed kinetic plasma simulations that included energized O^+^. The work presented here is motivated by observations of energized O^+^ in the magnetotail together with a lack of corresponding simulation studies of magnetic reconnection involving energized oxygen. Kronberg et al. (2014) gives an excellent overall review of the transport and acceleration (energization) of heavy ions in the magnetosphere and magnetotail.

The dawn‐dusk electric field across the magnetotail CS predicts cross‐tail ion acceleration as evidenced by an increased dusk‐side asymmetry of energized ions (Lyons & Speiser, 1982; Meng et al., 1981; Speiser, 1965). Several investigations of cross‐tail electric field acceleration of protons and O^+^ have been undertaken resulting in acceleration estimates of >50 keV O^+^ (Birn et al., 2004), 100–200 keV H^+^ (Birn & Hesse, 1994), 20 keV O^+^ (Ipavich et al., 1984), 50–500 keV H^+^ (Meng et al., 1981), and 112–157 keV O^+^ (Wygant et al., 2005). Without regard to the acceleration mechanism, energized O^+^ in the 12–40 keV range has been observed by Kistler et al. (2005) streaming dawn to dusk in the magnetotail at about 19 *R*_*E*_. While the streaming O^+^ is identified as nonadiabatic, there is no mention of the CS structure or presence of a bifurcation.

### 1.5 Preview of Our Work

The following sections report our studies of energized O^+^ in the magnetotail and its effect on magnetic reconnection. We previously reported the creation of a bifurcated current sheet (BCS) due to a single population of Speiser‐orbiting heavy ions (George & Jahn, 2020). Here, we focus on a thinning CS in the presence of energized O^+^ and its effect on the onset and evolution of magnetic reconnection. We use a simplified magnetotail system configuration with oppositely directed magnetic field lines above and below the central CS. This makes the cross‐tail potential, and thus the natural energization of ions, duskward. As such it is natural for an energized ion to travel along a Speiser orbit and for a population of energized ions to form a BCS.

In Section 2, we present the methodology followed to simulate the thinning CS with energized O^+^ present. This includes the simulation code we used, the setup and parameter space we cover, and the analysis methods we apply to the simulation outputs to understand the system behavior. After this, in Section 3, we present the results of these simulations, starting with our baseline runs, then runs with variable O^+^ energization, and finally runs with different O^+^ density and CS thickness. We began our study by comparing the results of our simulations to those of published kinetic simulation studies involving two‐species and three‐species magnetic reconnection with thermal background ions. With these comparisons validating our setup, we introduced energized O^+^ to the basic magnetotail model. Although a physical magnetotail model contains more complex features, we excluded any major *B*_*z*_ and guide field components for ease in isolating results. This simple magnetotail model is in keeping with those used in previous kinetic studies. We purposely kept the system configuration simple to aid in the examination of this new area of simulation research. Our goal was not to produce a definitive study but to lay foundations for and justify further investigations into the effects of energized O^+^ on magnetic reconnection. Then, in Section 4, we discuss what these simulations show and examine effects that result from our simulation environment and those that represent the physics involved. This includes the parameter space of varying energization and density of the O^+^ as well as the CS thickness.

## 2 Simulation Methodology

We performed three‐species, 2.5D, PIC simulations of a thinning CS leading to magnetic reconnection. Our simulations are similar in setup to PIC studies of thermal O^+^ found in Table 1, extending our previous studies of energized O^+^ forming a BCS over a thinning central H^+^ CS (George & Jahn, 2020).

**Table 1:** Simulation Runs Performed

Run	O^+^ energy^a^	Onset @ time	Box size
1	No O^+^	630	320 × 80
2	Thermal O^+^	1,422	320 × 80
3	Thermal O^+^	1,474	320 × 160
4	0.7 keV	1,615	320 × 80
5	3.5 keV	2,725	320 × 80
6	5.25 keV	3,698	320 × 80
7	6.125 keV	4,830	320 × 80
8	7.0 keV	6,207	320 × 80
9	7.875 keV	7,500	320 × 80
10	8.75 keV	9,539	320 × 80
11	10.5 keV	8,373	320 × 80
12	14.0 keV	5,162	320 × 80
13	17.5 keV	2,443	320 × 80
14	0.7 keV	2,560	320 × 160
15	3.5 keV	5,454	320 × 160
16	5.25 keV	14,937	320 × 160
17	6.125 keV	20,870	320 × 160
18	7.0 keV	24,601	320 × 160
19	7.875 keV	25,690	320 × 160
20	8.75 keV	22,143	320 × 160
21	10.5 keV	14,854	320 × 160
22	14.0 keV	7,194	320 × 160
23	17.5 keV	4,223	320 × 160
24	7.0 keV	1,783^b^	320 × 80
25	14.0 keV	3,311^b^	320 × 80
26	7.0 keV	No onset^c^	320 × 80
27	14.0 keV	5,600^c^	320 × 80
28	7.0 keV	1,860^d^	320 × 80
29	7.0 keV	4,890^e^	320 × 80
30	14.0 keV	2,810^e^	320 × 80

^a^ Peak O^+^ energy once Speiser orbits have formed.

^b^ Lower density (0.05 *n*_*o*_).

^c^ Thicker CS.

^d^ Thinner CS.

^e^ Higher grid resolution.

While not a physical representation of either the magnetosphere or the magnetotail, we refer to the simulations as being a magnetotail‐like system oriented in the Geocentric Solar Magnetospheric (GSM) coordinate system. Using the Harris equilibrium for a magnetotail‐like system follows our previous O^+^ PIC simulations (George & Jahn, 2020). The simulation region is oriented to correspond to the GSM *X*‐*Z* plane in the center of Earth's magnetosphere at *Y* = 0. *Y* corresponds to the GSM *Y* duskward direction and out‐of‐plane direction. Unless otherwise noted in Table 1, the CS thickness is 1.5 proton inertial lengths in thickness. For the three runs using different CS thicknesses, the thinner is 1.0 and the thicker is 2.0 proton inertial lengths.

Our simulations begin with a CS centered between two regions of plasma in antiparallel magnetic fields (antiparallel indicating here that there are no guide fields or major *B*_*z*_ components to the magnetic field). This is the well‐known, commonly used, Harris equilibrium configuration (Harris, 1962). This follows previous PIC simulations of 3‐species, O^+^, plasmas to reduce variability at early stages of energized O^+^ investigations (George & Jahn, 2020). Our simulations, listed in Table 1, contain a three‐species plasma representing electrons, protons, and heavy ions. Following our previous work, we modified the three‐species thermal plasma by adding a small duskward velocity component. We assume that energization stems from the cross‐tail electric field (Section 1.4), although this is not the only possible source of energization in the magnetotail. A single two‐species simulation (Run 1) and two, three‐species, simulations with thermal O^+^ (Run 2 and Run 3) serve as a baseline for comparisons to previous simulation studies.

### 2.1 PIC Code

Our investigation uses a 2.5D, fully electromagnetic, semi‐implicit PIC code described in detail in Hesse and Schindler (2001) and Hesse et al. (2001b). In a 2.5D simulation, the particle positions are calculated in 2D while the particle velocities, electric fields, and magnetic fields are calculated in 3D. Hence, the designation of 2.5D. This has been used extensively in previous plasma studies involving reconnection, and specifically in our precursor study of O^+^ BCS (George & Jahn, 2020). Simulations involving thin CS and magnetic reconnection have been performed extensively using PIC codes (Hesse & Birn, 2004; Hesse & Schindler, 2001; Hesse et al., 1999, 2001a; Karimabadi et al., 2011; Liang et al., 2016, 2017; Markidis et al., 2011; Shay et al., 2007; Tenfjord et al., 2019). An explanation of this code can be found in the precursor work and its references (George & Jahn, 2020). The same computational platform described there was used for this work with the addition of 24 TBytes of additional local hard drive space. All velocities from our simulations are normalized to the proton Alfvén velocity. While no specific values for the magnetic field or proton number density are used in these simulations, for an estimated 0.55/cm^3^ proton number density in a 10‐nT magnetic field, the proton Alfvén velocity is ∼296 km/s.

### 2.2 Simulation Setup

We use two simulation box sizes. The larger box is double the size of the smaller along the *z*‐axis, i.e., perpendicular to the CS. The larger box was used to remove boundary interactions identified in the smaller box simulations. The smaller box is 320 × 80 proton inertial lengths, with a computational grid size of 800 × 400 cells. The larger box is 320 × 160 proton inertial lengths with a computational grid size of 800 × 800 cells.

The boundary conditions have been set along the *x*‐axis and *z*‐axis, in keeping with PIC simulations referenced previously. No initial perturbation was used for any of our simulations. For particles, the *X* boundaries are periodic. Any particle exiting one side reenters the opposite side with the same velocity vector. The *Z* boundaries are specularly reflecting. Any particle exiting the ±*Z* boundary reenters at the same location but with the opposite *v*_*z*_. For the electric and magnetic fields, the *X* boundaries are periodic and continuous. The *Z* boundaries are simple, reflecting boundaries for the electric and magnetic fields. This is accounted for in the implicit integration performed in the field calculations. In postprocessing, scaling and normalization of simulation parameters is unit‐less. Lengths are normalized to the proton inertial length (*c*/*ω*_*pi*_), while time is normalized to the proton gyroperiod, i.e., one time unit equates to a proton gyroperiod. The ratio between the electron plasma and gyrofrequencies is 5:1. This establishes the relationship between *B*_*o*_ and *n*_*o*_ where *B*_*o*_ is the peak magnetic field strength in the surrounding bulk plasma and *n*_*o*_ is the initial peak density at the center of the CS. A uniform background with a density of 0.1 *n*_*o*_ was used for the H^+^ in all runs. A uniform background with a density of 0.1 *n*_*o*_ was used for the O^+^ in all runs except Run 20 and Run 21, which used a density of 0.05 *n*_*o*_. The electron to ion temperature ratio was set to 0.2 and *ω*_*pe*_/*ω*_*ce*_ set to 5.0. A computational time step of an inverse electron plasma frequency, *ω*_*pe*_Δ*t* = 0.1, is used.

### 2.3 Mass Ratios

In an ideal simulation, the relative masses of the species would reflect physical ratios. This is both computationally prohibitive and unnecessary. Our study uses *m*_*e*_:$m_{H^{+}}$:$m_{O^{+}}$ mass ratios of 1:25:250. Previous kinetic studies have shown that these mass ratios, while not physical, are more than sufficient to separate the mass effects of electrons and protons as well as to study oxygen dynamics. While physical mass ratios are not used in this investigation, the lighter ions represent protons, hydrogen or H^+^, and the heavy ions represent oxygen or O^+^.

Kinetic simulations using the same Harris equilibrium and *m*_*p*_/*m*_*e*_ mass ratios of 25:1, 180:1, and 1,836:1, revealed no effect on the larger‐scale phenomena (Ricci et al., 2002). The evolution of two‐species reconnection was nearly identical for *m*_*p*_:*m*_*e*_ of 9:1, 25:1, 64:1, and 100:1 (Hesse et al., 1999), with a *m*_*p*_:*m*_*e*_ ratio of 25:1 separating the relevant electron physics from the proton physics. The results of Markidis et al. (2011), who used physical masses, was in agreement with those of Karimabadi et al. (2011) and Hesse and Birn (2004), who used reduced mass ratios. All reported a separation of scale between the three‐species for studies of both prereconnection and postreconnection evolution.

### 2.4 O^+^ Energization

Previous simulation studies of reconnection involving O^+^ focused exclusively on thermal O^+^ (Hesse & Birn, 2004; Karimabadi et al., 2011; Liang et al., 2016, 2017; Markidis et al., 2011; Tenfjord et al., 2019). Other than baseline simulations with thermal O^+^, our simulations use O^+^ initial conditions that are distinctly different. We use O^+^ that is energized as opposed to thermal. Run 2 and Run 3 are initialized with a random thermal distribution of 0.5 in units of $\left[m_{p}{V_{A}}^{2}\right]$. Tenfjord et al. (2019) demonstrated no significant effect in reconnection rate between runs with background O^+^ temperatures of 200 eV and 1 keV. O^+^ energization is achieved by adding a uniform *v*_*y*_ distribution to this thermal energy upon initialization of the simulation. This velocity approximates the acceleration that would be introduced by the cross‐tail electric field. No initial *v*_*y*_ was applied to the corresponding electron population as might be expected. This was not necessary as the much lighter electrons were immediately accelerated by the oxygen. A corresponding electron current was verified to form within a few hundred time units after initialization.

Giving the O^+^ an initial velocity is nonphysical, yet it naturally places the entire population into Speiser orbits forming a realistic result, that of a bifurcated CS (George & Jahn, 2020). Energization values of the O^+^ in the system were chosen to be in a range that has been observed in situ, but has never been investigated via kinetic PIC simulation (Kistler et al., 2005). Energization of the O^+^ results in the formation of a BCS sheet that is essentially superimposed over the central CS (George & Jahn, 2020). O^+^ energization ranging from 0.7 to 17.5 keV was applied, resulting in velocities normalized to the proton Alfvén velocity, from 0.775 to 19.384 in units of $\left[m_{p}{V_{A}}^{2}\right]$. In each case, the simulation is allowed to evolve to the point at which magnetic reconnection is fully developed. Evolution of the magnetic reconnection in each case is compared to determine the overall effect on the system.

### 2.5 Reconnection Onset and Rate

Several features can be used to establish the point at which the onset of magnetic reconnection occurs in a simulation. Onset accompanies the formation of one or more *X*‐lines or *X*‐points. This indicates that magnetic field lines that were previously directed opposite one another (antiparallel) have begun to merge. This alters their fundamental topology and increases the overall *B*_*z*_ component of the magnetic field. Onset is also indicated by the formation of a reconnection electric field located at the center of this *X*‐line. Additionally, onset can be identified by the formation of an out‐of‐plane quadrupole magnetic field (*B*_*y*_). A common method for identifying *X*‐line formation is to calculate the amount of reconnected magnetic flux (reconnected flux or flux herein) or Φ(*t*) found in Equation 1. Initial magnetic field lines are oriented parallel to the *x*‐axis and as such have only *B*_*x*_ components. As an *X*‐line is formed, the field line topology changes such that there is an increase of *B*_*z*_. We use three parameters based on the reconnected magnetic flux: instantaneous, differential, and integrated flux

(1) $$\mathrm{Instantaneous}\;\mathrm{Reconnected}\;\mathrm{Flux}\;:\;\Phi \left(t\right)=\frac{\int_{x}|Bz\left(x,z=0,t\right)|\;dx}{\int_{x}dx}$$

The instantaneous flux, Φ(*t*) (Equation 1), is an indication of the current state of the system evolution at a given time. As the flux changes, reconnection or merging has progressed. This is a line integral along our 2D CS as opposed to a surface integral of flux and is done due to the 2D nature of our simulation box. Generally, Φ(*t*) is calculated between *O*‐points and *X*‐points (Hesse & Birn, 2004; Markidis et al., 2011). This is done to ensure an easily identifiable and repeatable region for analysis; however, in our study, multiple *X*‐points can occur simultaneously. In this case, Φ(*t*) is calculated by taking the integral of the |*B*_*z*_| component along the CS (Equation 1). Since this encompasses the entire simulation box, it is also easily identifiable and repeatable. Although Hesse and Birn (2004) and Markidis et al. (2011) used it, they did not require the use of the absolute value operator since the polarity of *B*_*z*_ remained constant between *X*‐point and *O*‐point. This allows our calculation to span regions of both positive and negative *B*_*z*_. Increases in Φ(*t*) indicate *B*_*x*_ field lines reconnecting into *B*_*z*_ field lines. Φ(*t*) generally increases over time; however, this is not always true. Decreases in Φ(*t*) indicate the opposite with *B*_*z*_ field lines reconnecting into *B*_*x*_ field lines. This occurs when islands dissipate or plasmoids coalesce. Even though Φ(*t*) is decreasing, reconnection is proceeding. Additionally, we normalize this value to the magnetic field scaling factor *B*_*o*_ *c*/*ω*_*pi*_. This method is easily adapted to different tearing modes and does not rely on locating a specific point in the inflow or outflow regions. To give numerical consistency and to allow for comparison between runs, Φ(*t*) was used as an indicator to establish the point of onset of reconnection. At each point in time, the magnitude of |*B*_*z*_| was integrated along the magnetic null (*z* = 0) for all cells along the *x*‐axis. Our actual calculations were made near *z* = 0, i.e., we included a small band around *z* = 0 to account for small variations in the CS location. This integral was then scaled according to the number of grid cells along the *x*‐axis of the simulation box. A numerical threshold value of 0.005 *B*_*o*_ was selected for the “point of onset” as a common point of comparison. The reconnected flux reaching this threshold is our definition for the point of onset, herein referred to as the time‐to‐onset. This value was chosen such that the reconnected flux increased monotonically (near the point of onset) after exceeding the threshold

(2) $$\mathrm{Differential}\;\mathrm{Reconnected}\;\mathrm{Flux}\;:\;\Delta \Phi \left(t\right)=\frac{\left|\Phi \left(t+\Delta t\right)-\Phi \left(t\right)\right|}{\Delta t}$$

The differential flux, ΔΦ(*t*) (Equation 2), is an indication of the rate at which the system is evolving. The ΔΦ(*t*) is the change in Φ(*t*) per unit time and is also referred to as the reconnection rate. There are several methods to determine the reconnection rate. These rely on the type of reconnection model assumed, specifically, the overall topology of the evolution. Methods include the use of the reconnection electric field or the inflow and outflow Alfvén speeds (Comisso & Bhattacharjee, 2016). These methods are generally dependent on selecting the correct location to perform calculations. Here, we use the most basic definition of the reconnection rate: the amount of magnetic flux reconnecting per unit time. We previously calculated the flux, Φ(*t*), which gives an indication of how much magnetic flux has transitioned between *B*_*x*_ and *B*_*z*_ field line components. The differential change of reconnected flux with respect to time, ΔΦ(*t*), is then the amount of reconnecting flux per unit time (Equation 2). The reconnection rate is an indication of how fast the system is evolving. We normalize the differential flux to the bulk magnetic field value and the proton Alfvén speed, *B*_*o*_ *ν*_*A*_/*c*. To calculate ΔΦ(*t*), we evaluate Φ(*t*) at two points in time and divide by this difference in time. For a common point of comparison, we took the difference between *t* = 1,000 and *t* = 1,010

(3) $$\mathrm{Integrated}\;\mathrm{Reconnected}\;\mathrm{Flux}\;:\;\Sigma \Phi \left(t\right)=\sum_{0}^{t}\Phi \left(\tau \right)$$

The integrated flux, ΣΦ(*t*) (Equation 3), is a proxy of the global effectiveness of the system evolution. It is representative of the total amount of flux converted via reconnection. As the value of Φ(*t*) increases, so does ΣΦ(*t*), indicating reconnection is progressing. Φ(*t*) generally increases over time; however, this is not always true. As the value of Φ(*t*) decreases, ΣΦ(*t*) also increases. This decrease corresponds to a reduction in the *B*_*z*_ component across the CS. Physically this can occur via the dissipation of an island in the reconnection outflow, or by the coalescing of plasmoids. Since magnetic reconnection is not a reversible process, any change in the *B*_*z*_ component (i.e., back to *B*_*x*_ component) constitutes an increase in overall energy transfer. ΣΦ(*t*) is normalized the same way as the Φ(*t*).

## 3 Results

Our work began by generating baselines of our simulation setup with backgrounds of both H^+^ (Run 1) and thermal O^+^ (Run 2 and Run 3). We performed a visual comparison of these baselines against expected behavior from published research of a thinning CS undergoing magnetic reconnection. We then performed 10 simulations (Run 4 through Run 13), with varying energization applied to the O^+^ background. The simulation box size was doubled in the *z*‐dimension to address a small but quantifiable boundary interaction in the smaller box. We performed 10 additional, otherwise identical, simulations (Run 14 through Run 23) using the larger simulation box. We finally performed five simulations (Run 24 through Run 28) with variations of O^+^ density and CS thickness to evaluate any effect on the system.

Based on the simulations we computed three analysis parameters, described in Section 2.5, based on the amount of reconnected magnetic flux. These parameters were instantaneous flux, Φ(*t*), differential flux, ΔΦ(*t*), and integrated flux, ΣΦ(*t*). Then, based on Φ(*t*) crossing a threshold value, we determined the time‐to‐onset for each simulation.

The results of these simulations and calculations are presented below. First, we present the examination of the two baseline simulations performed for visual baseline comparison. Next are observations made of two of the simulations, one performed at 7.0 keV and the other 14.0 keV. Then, we present the results of the reconnected magnetic flux calculations. Finally, we present the findings from the time‐to‐onset determination followed by a discussion of the results.

### 3.1 Two‐Species (no O^+^)

For the first baseline simulation of our investigation, we visually compared our simulation setup (Run 1) against a two‐species, proton‐electron simulation of a thinning CS. Our setup was based on the well‐known Geospace Environmental Modeling (GEM) Reconnection Challenge (Birn et al., 2001). This challenge produced an excellent array of comparable results of two‐species reconnection in a common configuration (Birn & Hesse, 2001; Hesse et al., 2001a; Otto, 2001; Pritchett, 2001; Shay et al., 2001). The GEM challenge included an initial finite perturbation in the CS to speed up the dynamics. The rationale for such an initial perturbation was to put the system in the nonlinear regime of magnetic reconnection from the beginning of a simulation (Birn et al., 2001). Run 1 began with the same GEM configuration but without the perturbation such that the CS would thin naturally. While this required longer simulation times, it allowed comparison with our configuration runs that added O^+^. As a result, in Run 1, one central and four smaller tearing instabilities formed in the central CS. The central *X*‐point was the only one which fully developed. The four smaller ones were swept out by the outflow jets of the larger central *X*‐point.

Results of our two‐species simulation (Run 1) were consistent with the simulations of published studies. The evolution described in Section 1.2 occurred as expected. It was characterized by a single primary *X*‐point, with a slow increase in reconnected magnetic flux and a generally smooth evolution.

**Figure 1:**
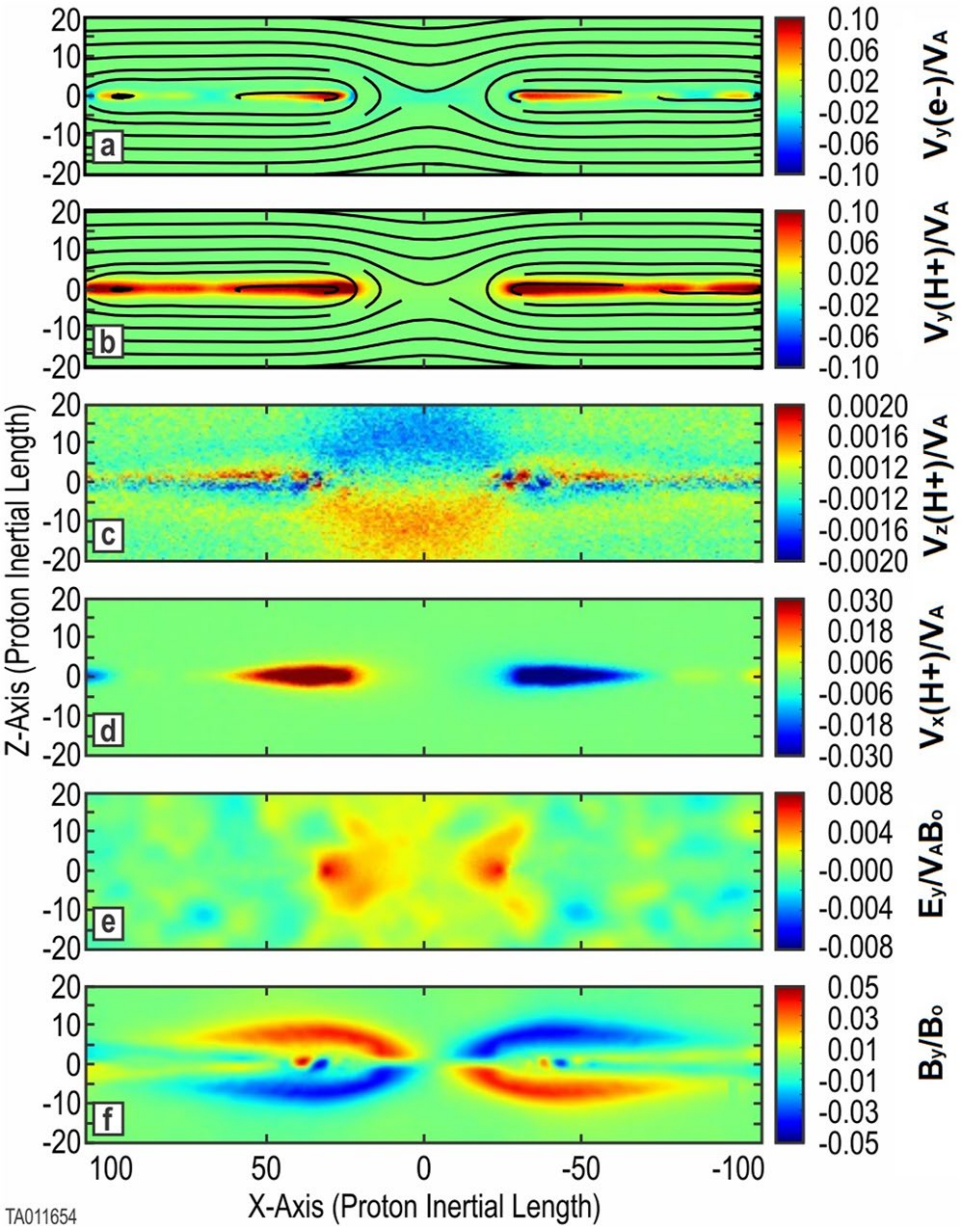
Key parameters for a two‐species simulation (Run 1). Shown are 2D color plots of key system parameters at *t* = 1,630 (onset of magnetic reconnection plus 1,000). Frames (a) and (b) are the *j*_*y*_ component of electron and proton current overlaid with the in‐plane magnetic field lines. Frame (c) shows the *v*_*z*_ component of the background protons. Frame (d) shows the *v*_*x*_ component of the protons forming outlet jets. Frame (e) gives the *E*_*y*_ component of the electric field. Frame (f) provides the *B*_*y*_ magnetic field.

Figure 1 shows the evolution and features confirming the expected results. Frames (a) and (b) show the formation of a primary *X*‐point where reconnection is taking place. There are also small secondary islands in the outflow jets forming smaller *X*‐point reconnection sites. Frame (c) indicates the inflow of background protons into the *X*‐point of the reconnection region. Frame (d) indicates the outflow jets of protons along the CS. Frame (f) shows the well‐defined out‐of‐plane (*B*_*y*_) quadrupole magnetic field. Since there were no indications of boundary interference, no equivalent of Run 1 was made in a larger box.

### 3.2 Three‐Species (Thermal O^+^)

We visually compared our baseline three‐species, (O^+^, H^+^, e^−^) simulation (Run 2) against published works (Hesse & Birn, 2004; Karimabadi et al., 2011; Liang et al., 2016, 2017; Markidis et al., 2011; Tenfjord et al., 2019). Results of our three‐species simulation with thermal O^+^ (Run 2) are consistent with published studies. Figure 7 (blue curves) shows a comparison of the difference in evolution between two‐species and three‐species (with thermal O^+^) simulations (Run 1 and Run 2). The time‐to‐onset, listed in Table 1, is delayed when O^+^ is added. There is also a small visible decrease in the rate of reconnected flux. This is in agreement with published results.

While there were no indications of boundary interference, an equivalent of Run 2 was performed in a larger box (Run 3). This additional baseline was performed to compare the large box simulation setup against Run 2 and the same published works mentioned above. The three‐species thermal O^+^ simulations (Run 2 and Run 3) evolved according to expectations in Section 1.2. The time‐to‐onset and reconnected flux profiles of Run 3 and Run 2 are nearly identical. Each run had multiple tearing instabilities forming in the CS prior to onset; however, only one primary *X*‐point ever developed.

**Figure 2:**
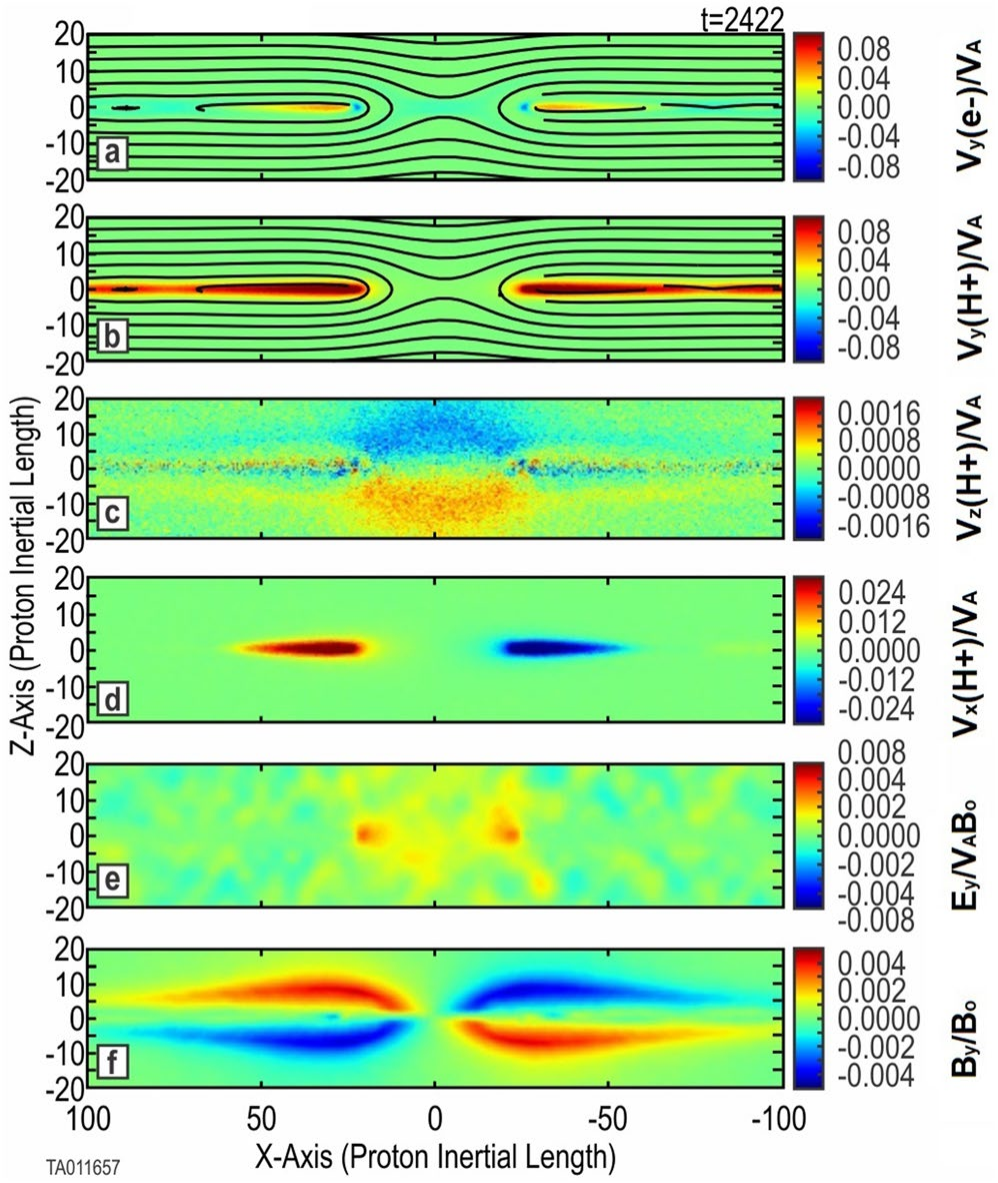
Key parameters for a three‐species simulation (Run 2) with thermal O^+^. Shown are 2D color plots of key system parameters at *t* = 2,422 (onset of magnetic reconnection plus 1,000. Frames (a) and (b) are the *j*_*y*_ component of electron and proton current overlaid with the in‐plane magnetic field lines. Frame (c) shows the *v*_*z*_ component of the background protons. Frame (d) shows the *v*_*x*_ component of the protons forming outlet jets. Frame (e) gives the *E*_*y*_ component of the electric field. Frame (f) provides the *B*_*y*_ magnetic field.

**Figure 3:**
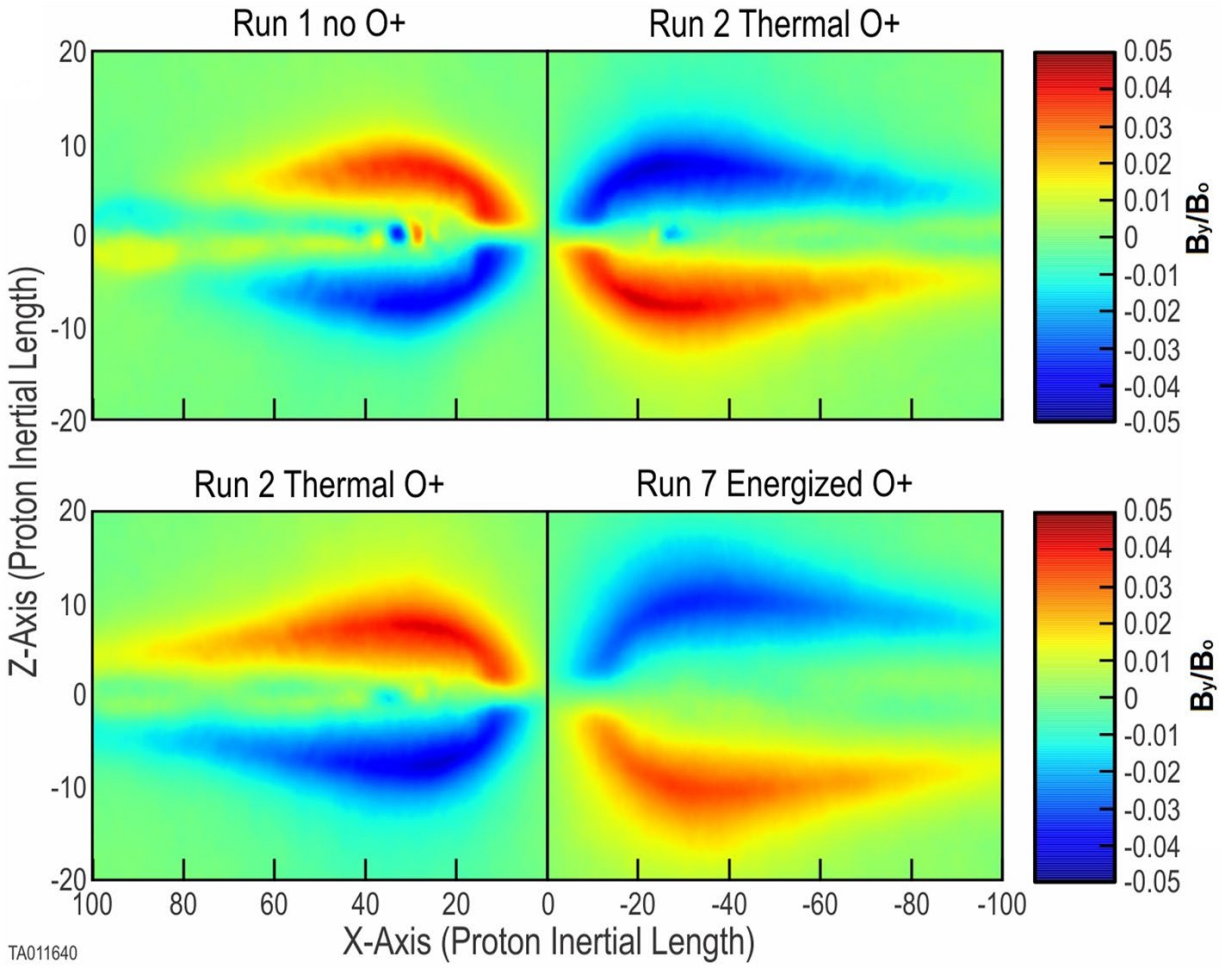
This shows color plots of the out‐of‐plane (*B*_*y*_) magnetic field produced aligned with the reconnection *X*‐line. These are all taken at 1,000 time units after the onset of their respective runs. On the top left is Run 1 with no O^+^ present, and on the top right is Run 2 with thermal O^+^ added. The larger scale produced by the thermal O^+^ is evident. On the bottom left is Run 2 with thermal O^+^ present and on the bottom right is Run 8 with 7 keV energized O^+^. The larger scale produced by the energized O^+^ is evident.

Figure 2 shows the evolution and key features, confirming the expected results at *t* = 2,422 (onset plus 1,000). Frames (a) and (b) show the formation of a primary *X*‐point where reconnection is taking place. Additional *X*‐points developed into small secondary islands in the outflow jets and did not support reconnection onset. We refer to these as islands to distinguish their minor effect compared to that of plasmoids, which have a major effect in evolution. Frame (c) indicates the inflow of background protons toward the *X*‐point of the reconnection region. A color plot of *v*_*z*_ for the O^+^ ions at the same time (not shown) indicates O^+^ ions are beginning to move toward the *X*‐point, although much more slowly. Frame (d) indicates the outflow jets of protons along the CS. The O^+^ outflow jets (not shown) are nearly indistinguishable from the H^+^ jets. Figure 3 shows a side‐by‐side comparison of the out‐of‐plane (*B*_*y*_) magnetic field between Run 1, with no O^+^, and Run 2, with thermal O^+^. For consistency each plot is taken at the time when instantaneous flux peaks (Figure 7). This gives an equivalent reference point in the evolution of each run. This comparison shows an increase in the scale size in the *X*‐direction. The broadened scale of the out‐of‐plane quadrupole structure has been reported previously by Shay and Swisdak (2004), Karimabadi et al. (2011), and Markidis et al. (2011), who show the *B*_*y*_ profile for their simulations without O^+^ and with thermal O^+^. Both indicate that the heavier mass of the O^+^ increases both the scale and intensity of the Hall effect quadrupole structure of *B*_*y*_.

### 3.3 Three‐Species (Energized O^+^)

The sections above discuss the baseline runs used to compare our configuration. The focus of this study is to investigate the effects of energized O^+^ on magnetic reconnection. Here, we present 25 runs that include a background of energized O^+^. These runs included 10 initial simulations (Run 4 through Run 13) and a second set of 10 runs in a larger simulation box (Run 14 through Run 23), with otherwise unchanged conditions. Additionally, these runs include five runs with variations in the O^+^ density and CS thickness (Run 24 through Run 28).

Examination of the simulations indicated that the systems were evolving according to two different topologies. We present a detailed description of two cases, 7.0 and 14.0 keV energization, representative of each topology. In both the small box and the large box simulations, the lower seven energizations exhibited a single primary *X*‐point where reconnection takes place. The higher three energizations of both simulation sizes evolved into a multiple *X*‐point plasmoid chain.

We present the effects of the energized O^+^ found by calculating the instantaneous, differential, and integral flux (Section 3.3.3) of each simulation. We also present our results from the determination of the time‐to‐onset of magnetic reconnection (Section 3.3.4), at each of various energizations.

#### 3.3.1 7.0 keV Energization

**Figure 4:**
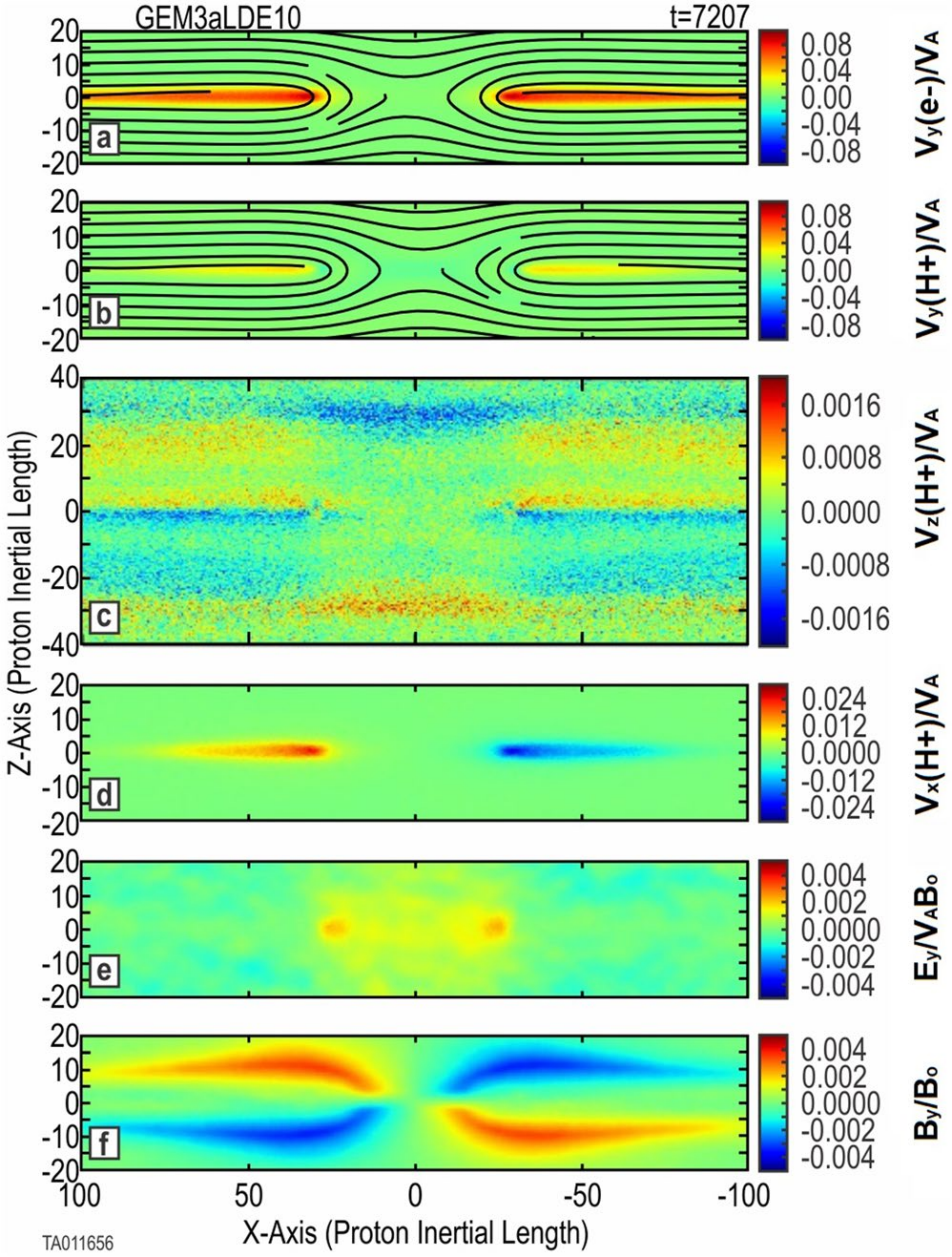
Key parameters for the 7.0 keV energized O^+^ simulation (Run 8). Shown are 2D color plots of key system parameters at *t* = 7,207 (onset of magnetic reconnection plus 1,000). Frames (a) and (b) are the *j*_*y*_ component of electron and proton current overlaid with the in‐plane magnetic field lines. Frame (c) shows the *v*_*z*_ component of the background protons. Frame (d) shows the *v*_*x*_ component of the protons forming outlet jets. Frame (e) gives the *E*_*Y*_ component of the electric field. Frame (f) provides the *B*_*y*_ magnetic field. A time sequence of the O^+^ bifurcated current sheet (BCS) is shown in Figure 6.

Several of the runs can be characterized by the formation of a single principle *X*‐point along the CS, leading to a slow, smooth evolution. Energizations from 0.7 to 8.75 keV (Run 4 through Run 10 and Run 14 through Run 20) fall into this category. The 7.0 keV simulation (Run 8) is typical and is described here. Figure 4 shows the key parameters of Run 8, allowing us to see that it evolves similar to the baseline simulations with no O^+^ and with thermal O^+^.

We note observations made from these simulations:

- The structure of the central CS, shown in frames (a) and (b), is the same as that of Run 1 and Run 2, with an *X*‐point forming in the center of the CS. The only major difference being the complete lack of any secondary island formation in the outflow regions.
- Frame (c) is significantly different than the baseline cases, showing a different evolution of the magnetic reconnection region. The inflow of H^+^, seen in Figures 1 and 2, is very near the CS at the *X*‐point. Frame (c) shows this inflow at ±30 proton inertial lengths, well away from the *X*‐point. The *v*_*z*_ color plot outside (±*X*) of the reconnection region, shows that H^+^ motion is away from the CS: red toward +*Z* and blue toward −*Z*. Additionally, along *Z* = 0, there is also H^+^ motion away from (or out of) the CS. As a result of this flow away from the CS, once reconnection has begun, this places the inflow further away from the central CS. This is in direct opposition to the H^+^ inflow seen in Figures 1 and 2.
- Frame (d) shows that the formation of H^+^ jets in the outflow is the same as in the baseline cases. For the baseline cases, the O^+^ jets were essentially the same as the H^+^ jets.
- This becomes distinctly different when the O^+^ is energized. Figure 6 shows the time evolution for the energized O^+^ in Run 8. This shows the *v*_*y*_ (left) and *v*_*x*_ (right) components of the bifurcated CS formed by the energized O^+^ at three different times. At time 6,000, prior to onset, the bifurcated configuration of the O^+^ is unperturbed. The two horizontal lines indicate the dual distribution of *v*_*y*_. Also, there is no significant *v*_*x*_ component. Inspection of individual particle traces (not shown) verifies that O^+^ moves in Speiser orbits gyrorotating between the upper and lower bulk regions around the central CS. The two *v*_*y*_ peaks (in orange) in the top left frame indicate where the O^+^ gyrorotates back toward the central CS. At time 8,000, the O^+^ ions have begun to turn outward. However, they are not limited to the same outflow region (near *Z* = 0) as the H^+^ seen in Figure 4. The *X*‐ward turning is revealed by the appearance of *v*_*x*_ components showing outward velocity. Time 10,000, outflow jets with a bifurcated structure similar to the original out‐of‐plane bifurcated structure in the central CS can be seen. Inspection of individual particle traces verified that the outward turning is occurring while the O^+^ Speiser orbits remain intact. The color plot of *v*_*y*_ changing to red indicates an acceleration of the O^+^ in *Y*, which is likely due to the Speiser orbits passing through the reconnection electric field.
- Frame (e) shows that the evolution of the reconnection electric field follows that of Runs 1 and 2.
- Frame (f) shows the development of a quadrupole out‐of‐plane magnetic field mirroring the structure of the *X*‐point.
- The bottom frames of Figure 3 show a side‐by‐side comparison of the out‐of‐plane (*B*_*y*_) magnetic field between Run 2 with thermal O^+^ and Run 7 with 7 keV energized O^+^. For consistency, each plot is taken at *t* = onset (as listed in Table 1) plus 1,000. This gives an equivalent reference point in the evolution of each run. This comparison shows an increase only in the scale size along the *X*‐direction, but also in the *Z*‐direction. There is also an increase in the spacing between the poles in the *Z*‐direction. This indicates an increased size of the reconnection region, not only beyond that with no oxygen, but beyond that with thermal O^+^.

#### 3.3.2 14.0 keV Energization

**Figure 5:**
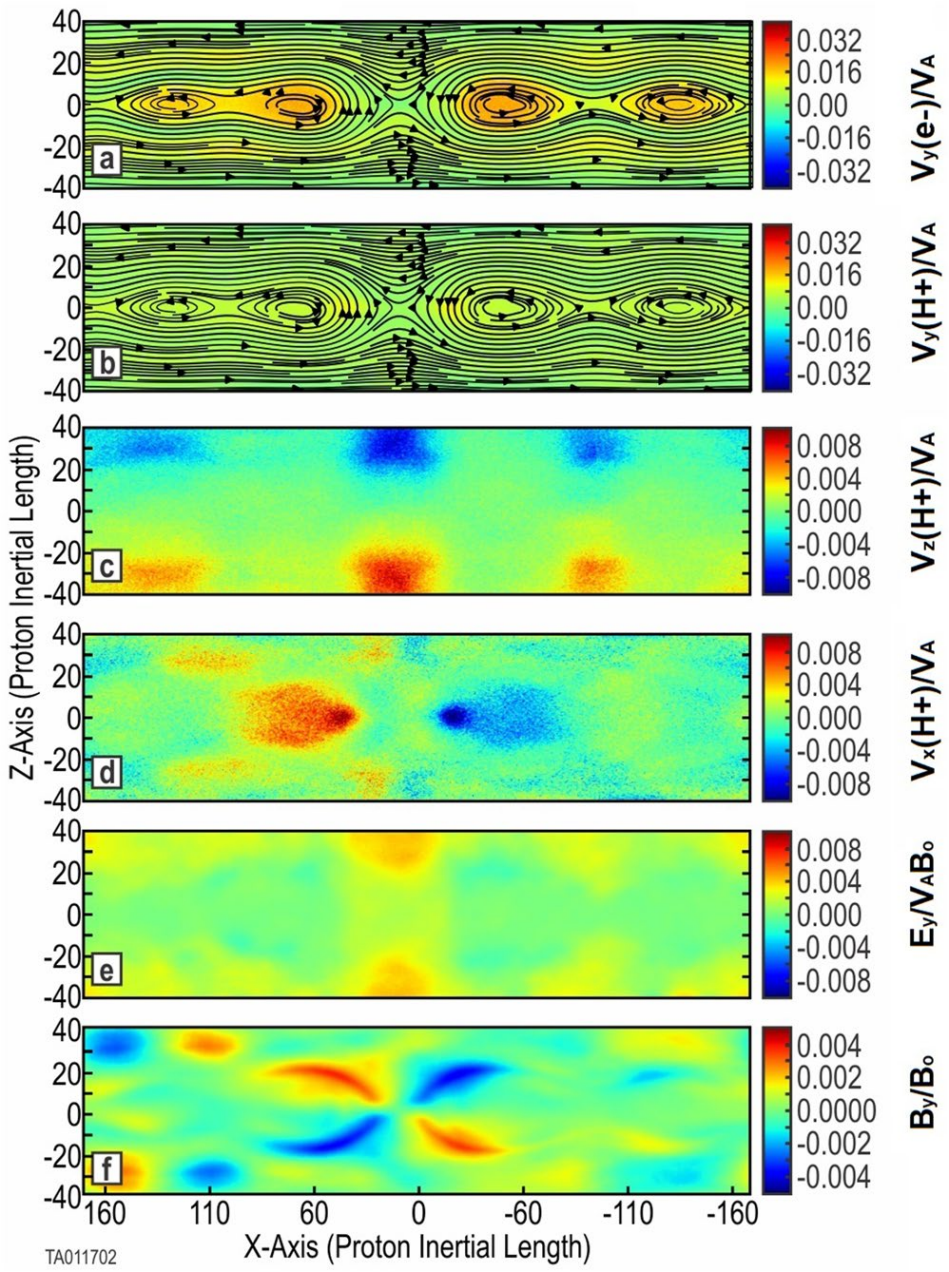
Key parameters for the 14.0 keV energized O^+^ simulation (Run 22). Shown are 2D color plots of key system parameters at *t* = 8,194 (onset of magnetic reconnection plus 1,000). Frames (a) and (b) are the *v*_*y*_ component of electron and proton current overlaid with the in‐plane magnetic field lines. Frame (c) shows the *v*_*z*_ component of the background protons. Frame (d) shows the *v*_*x*_ component of the protons forming outlet jets. Frame (e) gives the *E*_*Y*_ component of the electric field. Frame (f) provides the *B*_*y*_ magnetic field.

Several of the energized O^+^ runs can be characterized by the formation of multiple *X*‐points along the CS. Energizations from 10.5 to 17.5 keV (Run 11 through Run 13 and Run 21 through Run 23) belong to this group. The 14.0 keV simulation (Run 22) is representative of these simulations and is described here. The principal observation is that the CS is completely disrupted via a secondary tearing (plasmoid) instability. A detailed examination of the evolution of a stochastic plasmoid chain is beyond the scope of the present work. Markidis et al. (2012) provides a detailed study via PIC simulation of reconnection in a plasmoid chain. Figure 5 shows the key parameters of Run 22, allowing us to see that it evolves differently than both the runs similar to Run 8 and the baseline simulations with no O^+^ and with thermal O^+^. We note observations made from these simulations:

*X*‐lines began forming within the bounds of the central CS prior to reconnection onset. Numerous *X*‐points began forming and fully developed into plasmoids. Once onset was reached, the plasmoids had grown to be at least five times larger (in *Z*) than the initial CS thickness.

**Figure 6:**
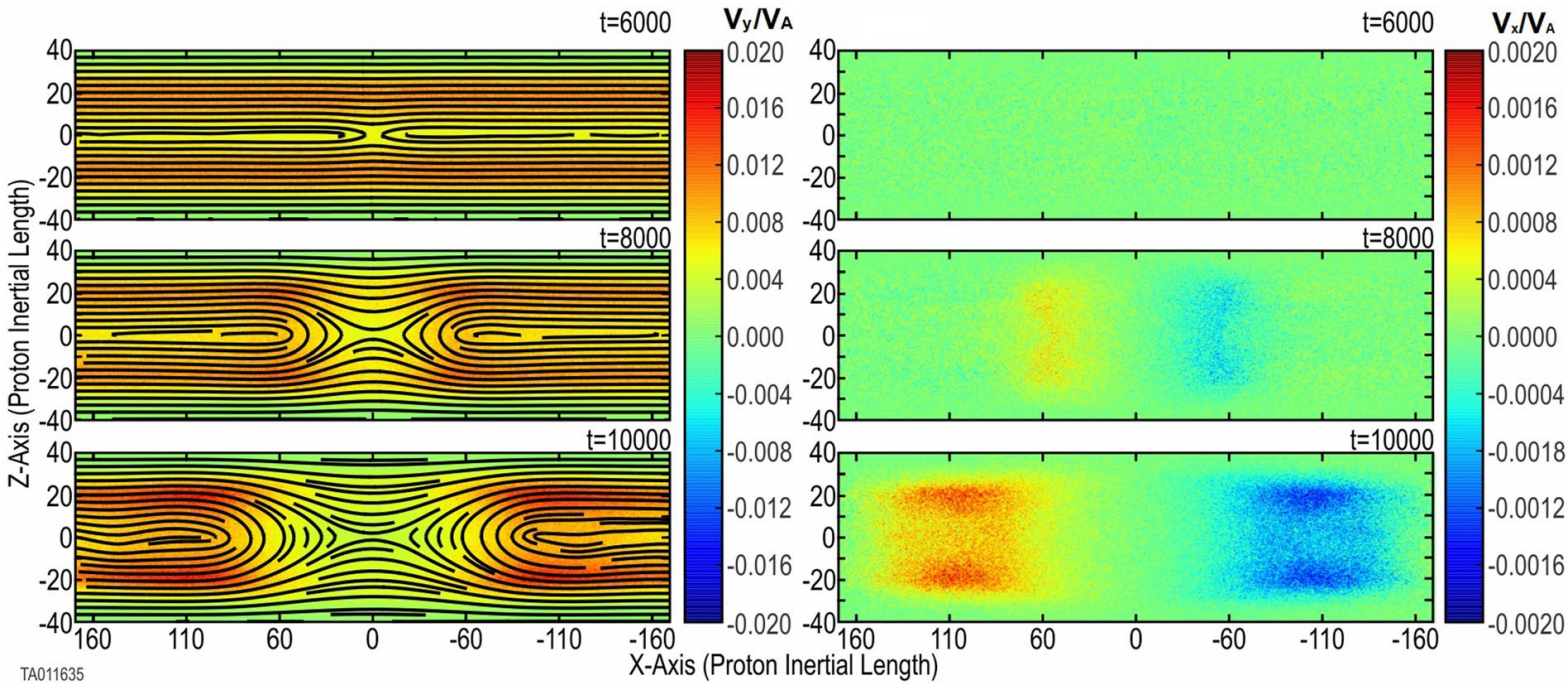
Time evolution of the onset of magnetic reconnection with a background of energized O^+^. Shown, top to bottom, is Run 8 at three times (6,000, 8,000, and 10,000). The left side shows the O^+^ velocity in the out‐of‐plane direction forming the duskward BCS overlaid with the in‐plane magnetic field lines. The right side shows the O^+^ velocity in the *X*‐direction, Earthward or tailward. This indicates the bifurcated O^+^ turning into the outflow region forming bifurcated outflow jets.

- Frames (a) and (b) show that the CS is completely subdivided and bounded by the plasmoids.
- Frame (c) shows motion toward the CS both above and below. This motion is associated with each of the *X*‐points
- Frame (d) shows H^+^ motion away from the *X*‐point. This motion is indicative of outflow jets. The jets are five times larger in the *z*‐direction than in Run 8 or the baseline runs. The evolution of the bifurcated CS (not shown) is similar to that of Run 8 shown in Figure 6, except that it is divided by the multiple *X*‐lines.
- Frame (e) shows that the evolution of the reconnection electric field is distinctly different than Run 8 or the baseline runs. For Run 8 and the baselines, the two points of localized *E*_*y*_ are on either side of the *X*‐point along the *x*‐axis. For Run 22, the two points of localized *E*_*y*_ are on either side of the *X*‐point along the *z*‐axis. This is also seen in Run 21, Run 12, Run 13, and Run 23. In Run 11, there were not two points of localized *E*_*y*_; instead it covered an area all around the *X*‐point.
- Frame (f) shows the development of one primary and multiple secondary quadrupole out‐of‐plane magnetic structures.
- The 10.5 keV (Run 11 and Run 21) were just beginning to develop multiple *X*‐points, but never fully developed into a plasmoid chain system during our simulations.
- The 14.0 keV (Run 12 and Run 22) simulations reached onset well before the previous three energies.
- The 17.5 keV (Run 13 and Run 23) simulations reached onset even sooner.

#### 3.3.3 Reconnected Magnetic Flux

We investigated the reconnected magnetic flux to understand the state, rate, and effectiveness of the reconnecting system evolution. We evaluated and compared the instantaneous flux, Φ(*t*), differential flux, ΔΦ(*t*), and integral flux, ΣΦ(*t*). These were calculated according to Section 2.5.

**Figure 7:**
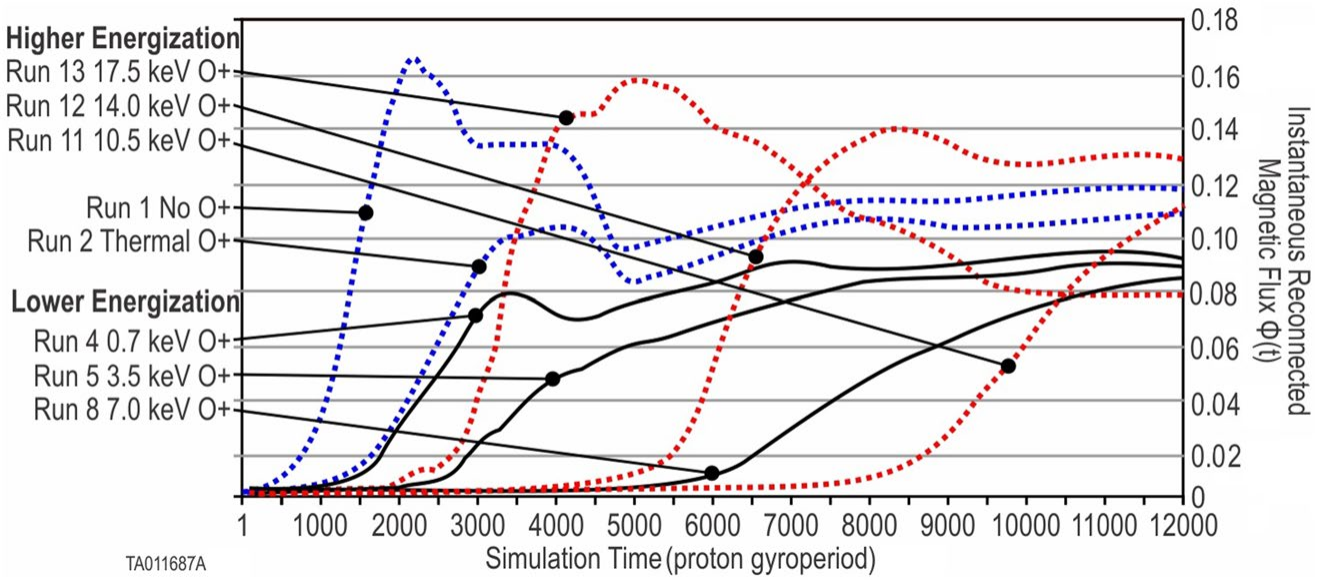
Instantaneous reconnected magnetic flux Φ(*t*): Taken from simulation time *t* = 0. Time plots of the instantaneous reconnected magnetic flux, Φ(*t*). The broken blue lines indicate two baseline simulations (Run 1 and Run 2). The three solid black lines indicate the lower‐regime simulations (Run 4, Run 5, and Run 8). The broken red lines indicate the higher‐regime simulations (Run 11, Run 12, and Run 13). The associated energization is indicated for each run. Φ(*t*) indicates the state of the system at a given time. Increasing amounts of Φ(*t*) indicate reconnection with the *B*_*x*_ magnetic field components converting into *B*_*z*_ components. Similarly, decreasing amounts of Φ(*t*) indicate secondary reconnection with the *B*_*z*_ magnetic field components converting into *B*_*x*_. This is indicative of coalescing of adjacent *O*‐points or plasmoids. Note that not all runs are shown in this plot.

Figure 7 shows plots of the instantaneous flux, Φ(*t*), plotted as a function of time. Φ(*t*) allows comparisons, between runs, of the overall evolution of the system. The broken blue curves show the baseline simulations, Run 1 and Run 2 for reference. Run 1 and Run 2 each show a peak then decrease of Φ(*t*). The decrease is due to secondary reconnection of the islands in the outflow region. These have the same relative behavior as the two‐species to three‐species thermal comparisons found in Karimabadi et al. (2011), Tenfjord et al. (2019), Markidis et al. (2011), and Liang et al. (2016). The thermal O^+^ in Run 2 caused a reduction (from no O^+^) in the peak amount of flux and the reconnection rate (slope). The solid black curves reveal the change in system response as energization increases. With 0.7 keV (Run 4) of energized O^+^, the peak Φ(*t*) and the reconnection rate (slope) were reduced slightly more than thermal O^+^. This is not unexpected, since this level of energization is only slightly higher than the thermal energy. Each subsequent increase in energization further decreases the reconnection rate and peak Φ(*t*). Run 11 sees an increase in both rate and peak. Although not within our simulation time frame, Run 11 would have likely experienced a peak as the slower‐forming plasmoids eventually coalesced. The broken red curves show a comparison of the difference in the general evolution for increasing levels of energization. Run 12 and Run 13 see an increasingly higher reconnection rate and peak Φ(*t*). Run 12 and Run 13 also show a peak then decrease of Φ(*t*). This decrease is due to secondary reconnection of the plasmoid chain.

While Φ(*t*) can increase or decrease depending on the occurrence of primary or secondary reconnection, ΣΦ(*t*) continuously increases over the system evolution. ΣΦ(*t*) is an indication of the overall effectiveness of the reconnection engine.

**Figure 8:**
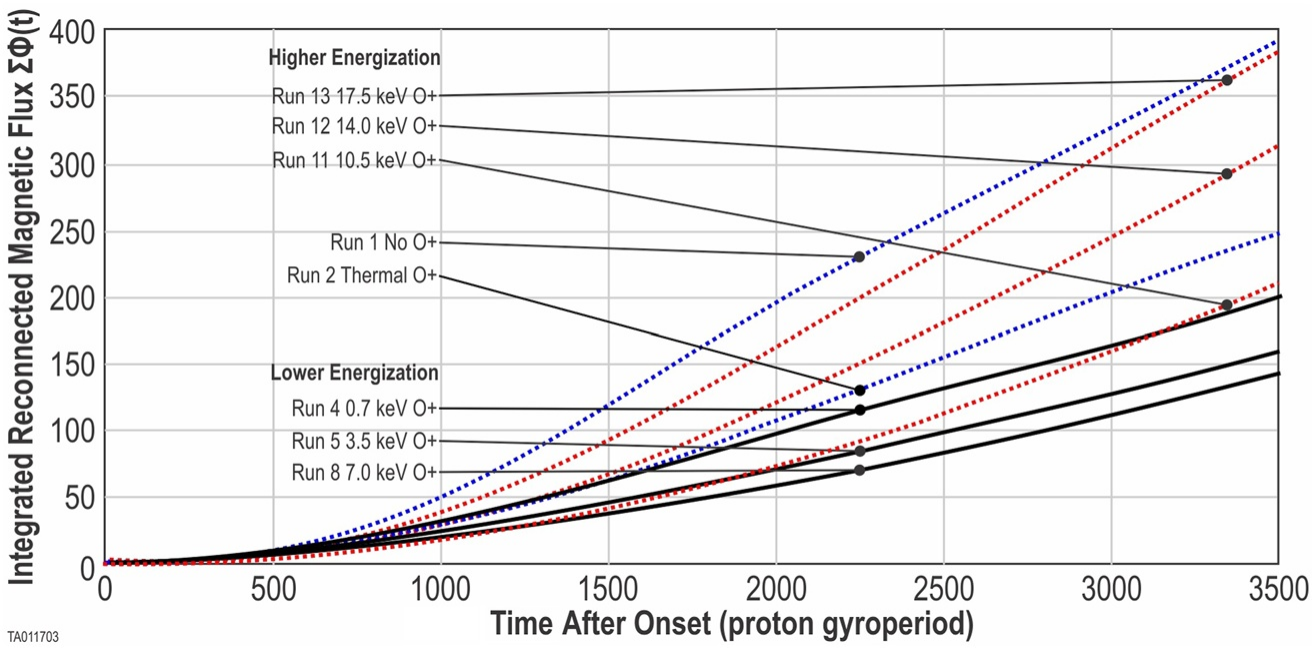
Integrated reconnected magnetic flux ΔΦ(*t*): Time is zeroed to start at the time of onset for each run. Run 1 is a baseline with no O^+^, Run 2 is a baseline with thermal O^+^, Run 4 has energized O^+^ 0.7 keV, Run 5 has energized O^+^ 3.5 keV, Run 7 has energized O^+^ 7.0 keV, Run 9 has energized O^+^ 10.5 keV, Run 10 has energized O^+^ 14.0 keV, Run 11 has energized O^+^ 17.5 keV. Note that not all runs are shown in this plot.

Figure 8 shows ΣΦ(*t*), the integrated flux, plotted as a function of time for each energization. This figure shows how the ΣΦ(*t*), and thus the overall effectiveness of the system, decreases with each increase for the lower energizations, then reverses and becomes more effective at the two highest energizations.

**Figure 9:**
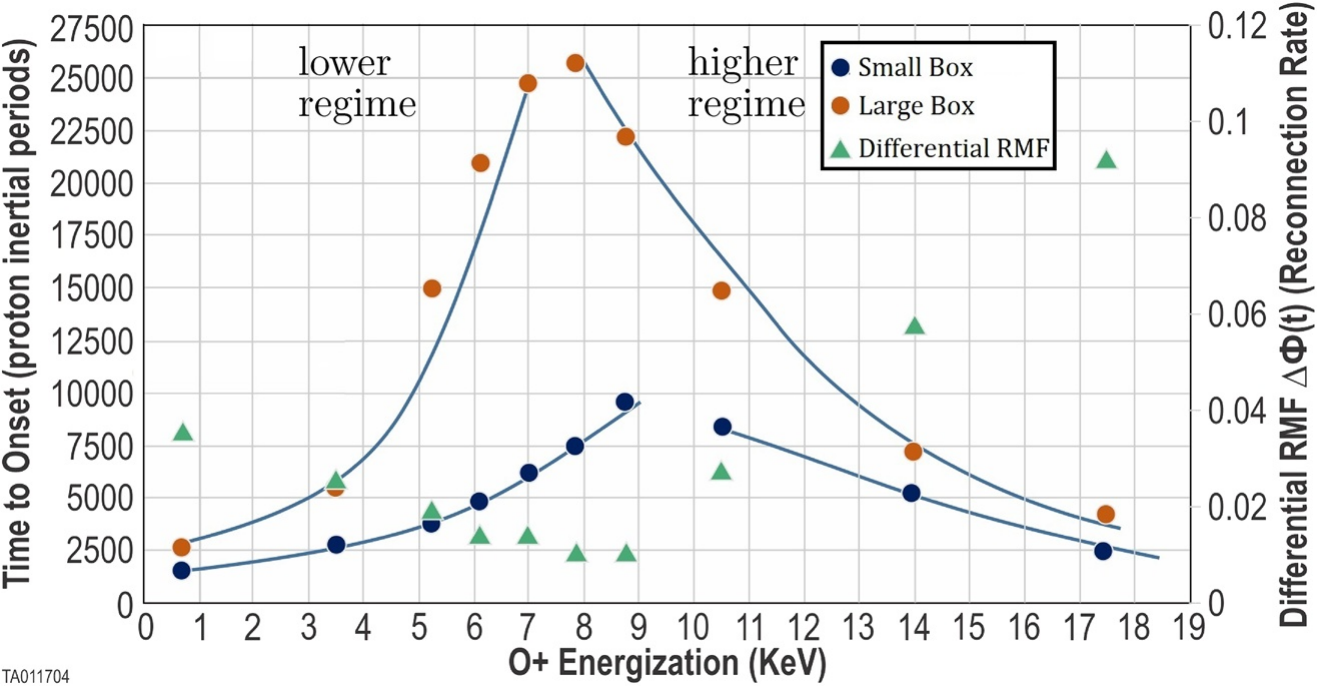
This figure depicts the lower and higher onset regimes for energized O^+^. The blue markers show the time‐to‐onset for the 10 O^+^ energizations in the small simulation boxes. The orange markers show the time‐to‐onset for the 10 O^+^ energizations in the large simulation boxes. The green markers show the differential flux for the 10 O^+^ energizations in the small simulation boxes. Differences in the time‐to‐onset and the point where the regime changes are discussed in Section 4.2.

To better analyze the variation in the reconnection rate, we calculated the differential flux, ΔΦ(*t*) for each energization. Values, at *t* = onset plus 1,000, are shown in Figure 9 (green triangle markers) using the right‐hand scale. As the energization increases at lower levels, the reconnection rate decreases. As the energization increases further, this trend reverses and the reconnection rate starts to increase.

#### 3.3.4 Time‐To‐Onset of Magnetic Reconnection

For a second comparison, we analyzed the time‐to‐onset of magnetic reconnection. Here, we found that O^+^ has a major impact. Each simulation was initiated at time zero with the same central CS thickness and same initial current profile. We ran 10 simulations (Run 4 through Run 13) with 10 values of O^+^ energization to cover the parameter space. Additionally, we ran 10 identical simulations (Run 14 through Run 23) using a larger simulation box. We determined the time‐to‐onset for each of these using the method in Section 2.5. These results are tabulated in Table 1 and plotted in Figure 9. Figure 9 shows the time‐to‐onset for the small simulation box runs (blue markers). As energization increases over the lower values, time‐to‐onset increases. When energization increases further an equally distinctive decrease is seen. Figure 9 also shows the time‐to‐onset for the large box simulations (orange markers). The same trends are observed in the larger simulation box; however, the trend reversal occurs at a lower energization. Also, the time‐to‐onset is systematically longer in the larger box. These differences are discussed in Section 4.2; which addresses the simulation box size.

### 3.4 O^+^ Density and CS Thickness

Two simulations with lower O^+^ density were performed at two energizations. Three simulations, with a thicker or thinner CS, were also performed at these two energizations.

Run 8 and Run 12 each had an O^+^ density of 0.1 *n*_*o*_. These were repeated with an O^+^ density of 0.05 *n*_*o*_ in Run 24 and Run 25, respectively. Run 8 reached onset at time *t* = 6,207, while Run 24 reached onset at time *t* = 1,783. Both reached onset with a single primary (*X*)‐line. A plot of instantaneous reconnected magnetic flux Φ(*t*) for Run 24 follows that of Run 4 in Figure 7 albeit shifted ∼100 to the right.

Run 12 reached onset at time *t* = 5,162, while Run 25 reached onset at time *t* = 3,311. Run 12 reached onset with multiple *X*‐lines, while the lower‐density Run 25 reached onset with a single primary *X*‐line. A plot of instantaneous reconnected magnetic flux Φ(*t*) for Run 25 follows that of Run 5 in Figure 7 albeit shifted ∼300 to the right.

Run 8 at 7.0 keV was replicated with a thicker and thinner CS in Run 26 and Run 28, respectively. While Run 8 reached onset at time *t* = 6,207, Run 26 with a CS thickness of 3.0 had not reached onset after time *t* = 25,000. Run 28 reached onset at time *t* = 1,860, well before Run 8. A plot of instantaneous reconnected magnetic flux Φ(*t*) for Run 28 follows that of Run 4 in Figure 7 albeit shifted ∼200 to the right.

Run 12 at 14.0 keV was replicated with a thicker CS in Run 27. Run 27 reached onset at time *t* = 5,600, nearly the same as Run 12 at time *t* = 5,162. A plot of instantaneous reconnected magnetic flux Φ(*t*) for Run 27 follows that of Run 12 in Figure 7 albeit shifted ∼400 to the left.

### 3.5 Background H^+^

An important aspect of the simulations is the different behavior of the H^+^ background between the runs with and without energized O^+^. All of the simulations started with an initial background population of thermal H^+^ equal to 0.1 *n*_*o*_. In all three baseline simulations without energized O^+^, this density remained constant at 0.1 *n*_*o*_ until reconnection onset. At that time, the background changed as it became involved in the reconnection inflow, as expected.

In contrast, during all of the runs with energized O^+^, the background H^+^ did not remain constant. Note that the O^+^ density profile remains relatively flat for all runs in this study as shown in Figure 3 of George and Jahn (2020). As time progressed, the H^+^ background was depleted near the CS and enhanced away from the CS.

**Figure 10:**
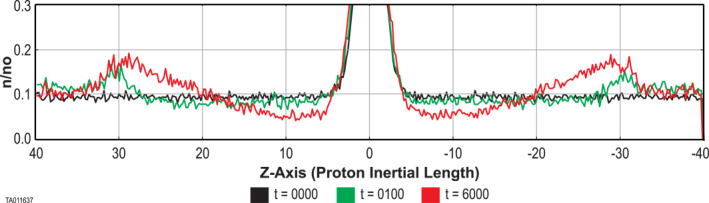
A time sequence plot of the H^+^ density in Run 8 at three points leading up to onset. The peaks are cut off only to emphasize the background population.

Figure 10 shows a time sequence of this depletion, which is typical of all of the energized O^+^ cases. With energized O^+^ present, the initial H^+^ density of 0.1*n*_*o*_ (black) immediately begins to deplete (green) near the central CS. There is also an increase in the H^+^ density above the initial value at about ±30 proton inertial lengths away. Just prior to onset the density around the central CS has depleted even further (red). The corresponding increase in the peaks at ±30 proton inertial lengths is even greater. This depletion became more pronounced as the O^+^ energization increased. We observed a difference in the depletion between simulations performed in the larger box and the smaller box, which is discussed in Section 4.2.

The profile of the *B*_*x*_ component of the magnetic field was unremarkable. In comparison to other energized O^+^ runs, there was effectively no difference up to the point of onset of magnetic reconnection. The Run 8 is the same simulation as Run 4 in George and Jahn (2020) where the *Z*‐cut profile over time is shown in Figure 4. Again, this profile evolution over time is nearly identical to the pre‐onset evolution of all other runs in this study.

## 4 Discussion

### 4.1 Two‐Regime Onset

In our simulations of a thinning CS, we see two distinct, system‐level response types to the onset of magnetic reconnection. These two response types manifest themselves in several ways: through differences in topology, the reconnected magnetic flux parameters (Section 2.5) and the time‐to‐onset. In otherwise identical simulations, these responses varied according to the energization of a background population of O^+^. Since these responses occur at either lower and higher O^+^ energization, we refer to them simply as the lower‐regime and the higher‐regime.

#### 4.1.1 Lower‐Regime

System responses in the lower‐regime follow a systematic evolution that is a function of increasing O^+^ energization. In the lower‐regime:

- Magnetic reconnection onsets via a tearing instability.
- The system forms a single primary *X*‐line.
- As energization increases:
  – The peak instantaneous reconnected flux decreases.
  – The differential (reconnection rate) reconnected flux decreases.
  – The integrated reconnected flux decreases.
  – The time‐to‐onset increases.

Given that CS thinning leads to the onset of magnetic reconnection, anything that alters this thinning must affect time‐to‐onset. A general mechanism for CS thinning in the magnetotail is the external lobe pressure applied to the central CS of the plasma sheet. This produces an imbalance of the external pressure to the internal pressure within the central CS. When the external pressure exceeds the pressure internal to the CS, the imbalance causes thinning of the CS, leading ultimately to onset of magnetic reconnection. It follows that a lessening of this external pressure would produce a reduction of the thinning. This in turn would produce a delay in reaching the onset of magnetic reconnection.

Our results indirectly show a difference in this external pressure between simulations with and without energized O^+^. Since the H^+^ background around the central CS is and remains thermal, its pressure is directly proportional to its density (*p* = *nk*_*B*_ *T*). All of our simulations were initialized with a thermal H^+^ background density of 0.1 *n*_*o*_. For Run 1 (no O^+^), Run 2, and Run 3 (both thermal O^+^), this background H^+^ density remained uniform at 0.1 *n*_*o*_. It remained so until onset, when the magnetic reconnection process began moving the background H^+^ ions away from the CS. This movement produced a distinct depletion around the CS and enhancement away from the CS (Figure 10). This depletion and enhancement occurred for all runs with energized O^+^.

Although the mechanism was not identified as part of this study, it is most certainly a direct result of the O^+^ energization. This depletion around the central CS directly reduces the external to internal pressure gradient between surrounding H^+^ and the central CS. This in turn slows the CS thinning, which ultimately delays of the onset of magnetic reconnection.

#### 4.1.2 Higher‐Regime

System response in the higher‐regime follows a systematic evolution, which is a function of increasing O^+^ energization. In the higher‐regime:

- Magnetic reconnection onsets via a tearing instability and is unstable to a secondary tearing (plasmoid) instability.
- The system forms multiple *X*‐lines.
- As energization increases above a critical transition:
  – The peak instantaneous reconnected flux increases.
  – The differential (reconnection rate) reconnected flux increases.
  – The integrated reconnected flux increases.
  – The time‐to‐onset decreases.
  – The number of plasmoids increases.

Transitioning from the lower to higher‐regime is evidenced by a major change in the reconnection topology, specifically, changing from a single primary *X*‐line to multiple *X*‐lines. The mechanism causing this transition is correlated with the presence of energized O^+^. In the lower‐regime, the O^+^ caused the H^+^ to move away and slowed the CS thinning. At the transition point between both regimes, O^+^ continues to affect the H^+^, moving it away from the CS. In fact, the higher the energization, the more effect it has on the background H^+^. Examination of the H^+^ depletion indicates that the effects in the higher‐regime occur as a result of this depletion.

In the lower‐regime, O^+^ moves the H^+^ away from the vicinity of the central CS. In the higher‐regime, the H^+^ depletion is more pronounced. This continues the correlation between increased O^+^ energization and increased depletion seen in the lower‐regime. This enhanced depletion causes a higher internal to external pressure gradient. Near the transition point between regimes, the depletion of H^+^ not only slows and stops the CS thinning, it reverses it, and the CS begins to broaden. It broadens so much that the particle and current density in the central CS begins to diminish. The out‐of‐plane current peak in the central CS also begins to diminish as it broadens. In comparison, this is evident by the color bar values (intensity) of Figures 5a and 5b being half of those of Figures 4a and 4b. As the CS broadens to approximately twice the original thickness, multiple *X*‐lines form within its bounds. This is referred to as a secondary tearing or plasmoid instability. There is no magnetic reconnection taking place as evidenced by the lack of a reconnection electric field or a quadrupole magnetic field. The amount of reconnected magnetic flux remains low and fluctuates. Once the point of onset of magnetic reconnection is reached, as determined by Φ(*t*), the plasmoid size exceeds the original CS thickness (Figure 5). Secondary reconnection begins, and the plasmoids eventually coalesce.

In addition to the diminishing content of the central CS, there could be an additional disruption mechanism caused by the Speiser‐orbiting O^+^. As the O^+^ has an effect on the background H^+^, it could also have an effect on the CS. The scope of this work did not include investigating such a mechanism.

In Figure 8, the ΣΦ(*t*) curve at 10.5 keV (Run 11) crosses the curve at 3.5 keV (Run 5) at approximately time *t* = 1,700 and the curve of 0.7 keV (Run 4) at approximately time *t* = 3,100. Looking at the topology of Run 11 over time (not shown), this run commences with two primary *X*‐lines and a third, not fully formed, *X*‐line. This third *X*‐line fully develops shortly after onset, increasing the reconnection rate ΔΦ(*t*) and boosting ΣΦ(*t*).

Examination of O^+^ density in Section 3.4 indicates that less O^+^ reduces the H^+^ depletion, the CS thinning and the time to reconnection onset. While not an exhaustive comparison, these two additional runs indicate that a decrease in number density produces a lesser effect. Note that Run 10 with 14.0 keV resulted in a faster onset time due to a different mode of instability disrupting the CS. Run 21, also with 14.0 keV but with 0.05 *n*_*o*_ O^+^ density, formed a single *X*‐point, indicating that a tearing mode became dominant.

Results in Section 3.4 indicate that the CS thinning and time to reconnection onset is dependent on initial CS thickness. A thicker CS, on the other hand, did not reach onset even after 25,000 proton gyroperiods.

Figure 5 shows that the reconnection electric field, *E*_*y*_, in Run 22 is distinctly different than what is seen in the lower‐regime or baseline runs. We expect that there are two points of localized *E*_*y*_ on either side of the *X*‐point along the *x*‐axis. For Run 22, the two points of localized *E*_*y*_ are on either side of the *X*‐point along the *z*‐axis. This variation in reconnection electric field location is seen in four out of six higher‐regime simulations. This is possibly due to the depletion of the H^+^, which may form Hall currents moving in a nontraditional inflow region toward the *X*‐point. A nontraditional inflow region refers to that seen in Figure 4c. It also may be caused by the two center plasmoids beginning to coalesce. In either case, it is beyond the scope of this present investigation and will require further study.

There is a critical O^+^ energization at which the system transitions from the lower‐regime to the higher‐regime. In our simulations, this occurs between 8.75 and 10.5 keV in the small box and between 7.0 and 7.875 keV in the large box. Why this happens is a result of simulation box size and is discussed in Section 4.2.

While this regime change is described as a combination between the differences in both the onset and evolution of magnetic reconnection, it is suggestive of a collisionless plasma transitioning across *λ*_crit_ as depicted in a reconnection phase diagram (Section 1.2; Ji & Daughton, 2011). The *λ*_crit_ separates regions where the onset of magnetic reconnection leads to a single *X*‐point or to multiple *X*‐points in a plasmoid instability. This appears to be what is happening in our system. However, the phase diagram referenced does not take into account effects due to energized heavy ions. Uzdensky and Loureiro (2016) also present two onset regimes: one that produces a single‐island and one that produces a multi‐island system. These two regimes are referred to as the Furth, Killeen, and Rosenbluth (FKR) or the Rutherford regime and the Coppi regime. Which regime is dominant is highly dependent on the rate of CS formation. In recent years, the unlikelihood of a Sweet‐Parker‐like CS forming due to background turbulence and the CS instability (Loureiro & Uzdensky, 2016 and references therein) has been discussed extensively. This does not preclude a region of high Lundquist number, *S*, and low effective plasma size, *λ*, where single *X*‐point, collisionless, reconnection can take place (Ji & Daughton, 2011). Additionally, as is seen in published thermal O^+^ studies and our lower‐regime, O^+^ can add stability to the CS. This is especially possible considering the exclusion of thermal or energized O^+^ in any associated studies of plasmoid instability. There also have been no investigations of the effect of energized O^+^ on the Lundquist number.

There is an interesting parallel in the similarity between our two‐regime system with energized O^+^ and those mentioned above. Our two‐regime response is due to an independently controllable variable applied to an otherwise invariant system. This provides an in‐depth method to study what causes a system, stable to a single *X*‐line, to transition to one that develops a plasmoid chain.

### 4.2 Simulation Size

Two anomalies were seen when comparing the small and large simulations. First, the time‐to‐onset values were systematically larger in the large simulation box. Second, the point at which the larger box transitioned from lower‐regime to higher‐regime was at a lower energization than the smaller box. Examination of the background H^+^ for each box at each energization reveals the mechanism leading to both of these anomalies. Based on this examination, we show that the physics simulated in the small box remains valid even though there are small *Z*‐boundary interactions.

The original box size for this study was selected to ensure that energized O^+^, in Speiser orbits, would not interact with the boundaries of the simulation. We previously showed that a sustained O^+^ BCS can be simulated in the original box without experiencing boundary interactions (George & Jahn, 2020). This box was computationally 800 × 400 grid cells (*X*‐dimension × *Z*‐dimension) and physically 320 × 80 proton inertial lengths. During the course of the investigation, we found that there was a small, but quantifiable, boundary interaction. When the energized O^+^ displaced the background H^+^, the displacement extended beyond the ±Z range of the O^+^ and into the ±Z boundaries. Due to this interaction, we enlarged the box and reperformed the simulations. For these, we doubled the size of the box in the *Z*‐dimension. This resulted in a larger box that was computationally 800 × 800 grid cells and physically 320 × 160 proton inertial lengths.

Since there was absolutely no boundary interaction with Run 1, we did not reperform it in the larger box. Since the thermal O^+^ was used to baseline the energized runs, Run 2 (thermal O^+^) was replicated in the larger box in Run 3. Both reached reconnection onset at essentially the same time, *t* = 1,422 and *t* = 1,474, respectively. Each evolved in a nearly identical manner after reaching onset, which indicates that there was no interaction due *Z* boundaries in the smaller box.

For the energized O^+^ runs in the smaller box, we noted a small, but quantifiable boundary interaction that warrants discussion. To address and determine the extent of the interaction on our results we reran these simulations using a larger box (320 × 160).

**Figure 11:**
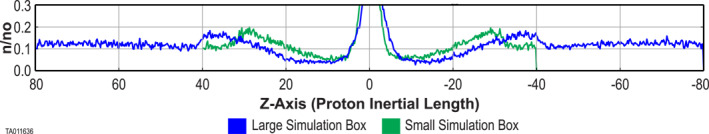
*Z*‐cut of H^+^ density at the center of the simulation box, just before onset, for Run 8 (small simulation box—green) and Run 18 (large simulation box—blue). Both are taken at time *t* = 6,000. This shows that the *Z* boundary at ±40 lessened the H^+^ depletion (green) around the central CS when compared to that in the larger simulation. The larger box allows for H^+^ depletion to extend a few gyroradii further. This lowers the density immediately around the central CS, reducing the thinning and further delaying the offset.

The same energized O^+^ populations were used in these simulations and, again, had no boundary interaction. As discussed above, the Speiser‐orbiting energized O^+^ caused a depletion region to form in the background H^+^ around the central CS. While depletion occurred near the central CS, peaks were formed at the edge of the bifurcated CS with the displaced H^+^. Figure 11 shows that the locations of the peaks around the BCS differ slightly between otherwise identical simulations in the small and large box. For Run 8 (smaller box), these peaks occurred at about ±30 proton inertial lengths. For Run 18 in the larger box, these peaks occurred at about ±38 proton inertial lengths. Except for the simulation box size, each set of 10 simulations were otherwise identical. Each of the 10 runs with varying energization was replicated in a larger simulation box. In each case, the time‐to‐onset was greater in the larger box (Figure 9). In each case, an examination of the H^+^ density depletion around the central CS reveals the source of this interaction. The boundary of the smaller box actually prevented the background H^+^ from moving further out. This is due to the plasma back‐pressure against the specularly reflecting *Z* boundary. Once the box was enlarged, this back‐pressure no longer hindered the outward movement of the background H^+^. This allowed the background around the central CS to deplete an additional amount, which further reduced the external pressure on the central CS. This slight reduction was enough to further slow the thinning and extend the time‐to‐onset. This explains the increased time‐to‐onset as the energization increased. It also explains why the system transitioned from the lower‐regime to the higher‐regime. Very simply, between the larger box and the smaller box, Figure 11 shows that the H^+^ needed just a little more room to be pushed out just a little bit farther. Preventing the H^+^ from being pushed out that small amount was enough to increase the density around the central CS; producing enough external pressure to thin the CS, which initiated onset sooner in the smaller box. Similarly the external pressure was enough to balance the CS until a slightly higher energy was reached, sufficient to produce the transition to the higher‐regime in the smaller box. In the larger box, it is evident that the additional density reduction slows the CS thinning even more, producing a much greater onset delay. From these comparisons of the large to small simulations, we can conclude that even though the smaller box did affect the quantitative results, qualitatively the physics (of the background H^+^ depletion and the CS disruption) is the same in the smaller box.

### 4.3 Grid Resolution

Simulations 1 through 28 were performed with grid cell resolutions resulting in one electron inertial length per cell (in *Z*) and two electron inertial lengths per cell (in *X*). In order to investigate possible effects related to numerical resolution, we reperformed runs 8 and 12, as 29 and 30, with the grid resolution doubled in both *X* and *Z*. We maintained the same small physical box (320 × 80 proton inertial lengths); however, we used a higher grid resolution (3,200 × 1,600). We limited the number of runs at this enhanced grid resolution since each simulation took more than 2 months of computational time. The two simulations we ran confirm the same evolution with cell sizes of 1/16 the size of the original runs. This is equivalent to grid cell resolutions of four electron inertial lengths (in *Z*) and eight electron inertial lengths per cell (in *X*). This should be sufficient to prevent introduction of numerical noise into the calculations.

Run 29 at 7.0 keV reached onset sooner at time 4,890 compared to 6,207 for Run 8. Run 30 at 14.0 keV also reached onset sooner, at time 2,810, compared to 5,162 for Run 12. While both simulations at the higher grid resolution reached onset sooner than their corresponding simulation at lower resolution, the qualitative response, system evolution, and onset regime, followed the same two‐regime onset and development as the lower grid resolution simulations. Run 29 developed into a single *X*‐line characteristic of the lower‐regime as did Run 8. Run 30 exhibited the same multiple *X*‐line, i.e., plasmoid instability, as did Run 12 in the higher‐regime.

The scope of this work did not include a detailed analysis of the differences between the higher and lower resolution simulations. A more thorough examination of these differences is warranted. We suggest that a study be undertaken with intermediate grid resolutions and a close comparison of the various parameters, not just time‐to‐onset.

## 5 Summary and Conclusion

The study of O^+^ in reconnection simulations has been limited to a background of thermal ions. Published results demonstrate that the effects of thermal O^+^ on magnetic reconnection are relatively minor. Thermal O^+^ essentially behaves like heavier protons. To study the effect of energized O^+^ on the magnetic reconnection process, we first baselined our simulation setup, repeating published simulations with a background of either thermal H^+^ or thermal O^+^. Our results mirrored those of published results, indicating a valid setup.

Since energized O^+^ has been observed in conjunction with CSs and magnetic reconnection, it should be examined in simulation studies. We introduced a population of energized O^+^ into our baseline simulations of a thinning CS. We ran 25 simulations with variations in O^+^ energization, density, and CS thicknesses. We analyzed the evolution and onset of each system by comparing key parameters of each simulation. These included the CS structure, the inflow and outflow region structure, and the out‐of‐plane electric and magnetic field formation. We studied the effects of energized O^+^ by analyzing the instantaneous, differential, and integral flux (Section 3.3.3). This provided information about the state of each system, the rate at which it evolved, and its overall effectiveness as a reconnection engine. Finally, we determined the time‐to‐onset of magnetic reconnection (Section 3.3.4) at the various energizations. From these results, we can conclude that:

- Energized O^+^ has a major impact on the onset and evolution of magnetic reconnection.
- The presence of energized O^+^ causes a two‐regime onset response in a thinning CS.
- At lower energization, O^+^ increases time‐to‐onset and suppresses the rate of evolution.
- At higher energization, O^+^ decreases time‐to‐onset and enhances the rate of evolution.

Our results show that, unlike thermal O^+^, energized O^+^ populations do have a major impact on the onset and evolution of magnetic reconnection. Changes in both the energization and number density of the O^+^ contribute to its impact. Over the energy range we studied, we found that energized O^+^ leads to a dual‐regime response of these parameters. These regimes are based on O^+^ energization, and are referred to as the “lower‐regime” and the “higher‐regime.”

In the lower‐regime, the time‐to‐onset of reconnection increases with O^+^ energization, while the amount of reconnected flux and reconnection rate decrease. Similarly in the higher‐regime, the time‐to‐onset of reconnection decreases with O^+^ energization, while the amount of reconnected flux and the reconnection rate increase.

The resultant topologies in these two regimes show that magnetic reconnection proceeds according to two different mechanisms. Although their evolution is quite different, they both appear to be a result of tearing instabilities in the CS. In the lower‐regime, reconnection occurs via a simple tearing instability at a single primary *X*‐point. In the higher‐regime, reconnection occurs at multiple *X*‐points, forming a stochastic plasmoid chain. Based on the concept of a “Phase Diagram” (Ji & Daughton, 2011), and given that a magnetotail plasma is collisionless and of high Lundquist number *S*, the system size is the dominant parameter that determines single or multiple *X*‐line evolution. For the study presented here, system size is consistent across simulations and would appear to have no effect on the evolution of reconnection. This would lead to the possibility that due to the presence of energized O^+^, some other parameter is affecting the evolution or that the O^+^ is producing a localized variation in the system size.

Closer examination of the evolution of both the lower and higher‐regimes shows the mechanism that causes the behavior. The Speiser‐orbiting O^+^ depletes the background H^+^ bordering the central CS. This H^+^ depletion around the central CS lowers the external pressure responsible for thinning the CS. The lower pressure slows CS thinning, leading to an increase in time‐to‐onset of magnetic reconnection. This effect is more pronounced as the O^+^ energization increases. Once onset occurs, depletion of H^+^ around the CS also starves the reconnection process of inflow material, slowing the evolution of reconnection itself.

In the higher‐regime, the same depletion drives the behavior. As O^+^ energization increases in the lower‐regime, it revealed that the CS thinning slowed. When energization reaches and exceeds a critical value, the CS not only stops thinning, but the process actually reverses, and the CS begins to broaden. It broadens enough that the particle and current density in the CS begin to diminish. This eventually disrupts the CS via an extensive tearing instability, referred to as a secondary tearing or plasmoid instability. This effect is more pronounced as the O^+^ energization increases, thus decreasing the time‐to‐onset. The creation and growth of multiple plasmoids facilitates numerous *X*‐points, driving the amount and rate of reconnected flux higher.

The two‐regime nature of the impact of energized O^+^ on tail‐like reconnection is a robust result over the parameter space considered. Future steps could include the study of energized O^+^ in 3D, and the inclusion of a physics‐based acceleration mechanism of O^+^ energization rather than an ad‐hoc seeding of the energized O^+^ population. Nevertheless, the behavior and contribution of energized O^+^ upon magnetic reconnection needs to be investigated in more detail to come to a full understanding of reconnecting systems under the influence of O^+^.

## Data Availability

Follow this link: https://zenodo.org/record/3593343#.Xiu_82hKi70 for the simulation code used in this study.
